# Large‐Scale Bioprinting of Human Epiblast‐Like Models Featuring Disc‐Shaped Morphogenesis and Gastrulation Events

**DOI:** 10.1002/advs.202505340

**Published:** 2025-06-05

**Authors:** Yixue Luo, Liheng Luo, Ling Wang, Shanshan Yang, Hongan Ren, Shaojun Liang, Xiaoyu Wang, Yijun Su, Leqian Yu, Xiaoyue Wang, Mingen Xu, Rui Yao

**Affiliations:** ^1^ Biomanufacturing and Rapid Forming Technology Key Laboratory of Beijing Department of Mechanical Engineering Tsinghua University Beijing 100084 China; ^2^ Center for bioinformatics National Infrastructures for Translational Medicine Institute of Clinical Medicine & Peking Union Medical College Hospital Chinese Academy of Medical Sciences and Peking Union Medical College Beijing 100730 China; ^3^ Key Laboratory of Medical Information and 3D Bioprinting of Zhejiang Province Hangzhou Dianzi University Hangzhou 310018 China; ^4^ Key Laboratory of Organ Regeneration and Reconstruction State Key Laboratory of Stem Cell and Reproductive Biology Institute of Zoology University of Chinese Academy of Sciences Chinese Academy of Sciences Beijing 100101 China; ^5^ Beijing Institute for Stem Cell and Regenerative Medicine Beijing 100101 China; ^6^ Institute for Stem Cell and Regeneration Chinese Academy of Sciences Beijing 100101 China; ^7^ School of Medicine Tsinghua Medicine Tsinghua University Beijing 100084 China; ^8^ Human Organ Physiopathology Emulation System Institute of Zoology Chinese Academy of Sciences Beijing 100101 China

**Keywords:** bioprinting, disc‐like epiblast models, human induced pluripotent stem cells, physical confinement, well‐defined bioink

## Abstract

Understanding the initial weeks of human development remains challenging due to ethical concerns and the restricted availability of human embryos. Pluripotent stem cell (PSC)‐derived epiblast models mimicking gastrulation processes have sparked significant interest in bridging this gap. However, as a newly emerging field, bioengineered models show limited production throughput and complexity in recapitulating epiblasts’ disc‐shaped morphogenesis. Here, a well‐defined laminin/alginate bioink to create epiblast‐like models from human induced pluripotent stem cells (hiPSCs) using electro‐assisted bioprinting is proposed. This approach enables the generation of large‐scale hiPSC‐laden microgels that not only facilitate mass transfer but also mimic the structural characteristics of early embryos, which allow hiPSCs to self‐organize into disc‐like epiblast models with consistent morphology and phenotype. With adaptability to human embryonic stem cells, this method demonstrates the versatility of engineering reproducible epiblast‐like models using various PSC lineages. Importantly, the bioactive components and physical confinement provided by the bioink, and the endogenous regulation of the WNT signaling pathway, contribute to disc‐like morphogenesis, recapitulation of epithelial‐to‐mesenchymal transition critical in the gastrulation process, and generation of the posterior epiblast population, directing the mass production of manipulable embryonic models for studying the spatiotemporal events and possible defects in early human development.

## Graphical abstract

Large‐scale generation of disc‐shaped models mimicking human epiblast layer is achieved by bioprinting of human pluripotent stem cells using a well‐defined biomatrix. The bioactive component and physical confinement from laminin/alginate microgels, combined with the endogenous regulation of the WNT signaling pathway regulated disc‐like morphogenesis, gastrulation‐stage epithelial‐to‐mesenchymal transition pattern and formation of early embryo models resembling the posterior epiblast population.

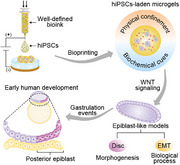

## 1 Introduction

The earliest stages of human embryo development initiate the dance of life but are hidden inside the mother. With dramatic morphogenesis happening from a ball of cells (known as the blastocyst) to a disc‐shaped cellular structure (known as the epiblast), giving rise to all three germ layers and forming future organisms,^[^ ^1^ ^]^ little is known about human developmental mechanisms. Human embryo culture complying with the globally agreed 14‐day rule partially solves this problem. However, human embryo availability and ethical constraints restrict extensive experimental investigation. The establishment of stem cell‐based models has attracted significant attention for deciphering the fundamental basis of a repeatable and manipulable system. These models could also help to understand the mechanism of birth defects and drug safety during pregnancy.

The year 2023 has witnessed a flurry of milestone reports on integrated human embryo models containing epiblast and extraembryonic tissues.^[^ ^2^, ^3^, ^4^ ^]^ These integrated models have been established by generating and co‐culturing embryonic and extraembryonic lineages through biochemical induction or gene editing^[^ ^2^, ^3^ ^]^ to mimic a key week of human development from peri‐implantation to peri‐gastrulation. Although these structures enrich the toolbox of embryo models, they raise profound ethical and regulatory concerns due to their similarity to intact human embryos in cell‐lineage composition and spatially organized morphogenesis.^[^ ^5^ ^]^ Therefore, carefully defining the scientific questions is essential to establish the most appropriate human embryo models for specific purpose. Non‐integrated stem‐cell‐based models mimicking some aspects of embryo development provide suitable alternatives, posing much fewer ethical challenges.^[^ ^5^ ^]^

The human epiblast layer is a sheet of pluripotent cells, presenting the disc‐shaped morphology and organizing into the three primary germ layers during gastrulation, which defines the basic body plan and eventually develops into all the organs.^[^ ^6^ ^]^ Non‐integrated models of the human epiblast offer a promising avenue for investigating developmental events during post‐implantation.^[^ ^7^, ^8^, ^9^ ^]^ These epiblast‐like models, exhibiting cavity structure instead of disc‐shaped morphology, are generated by embedding human pluripotent stem cells (PSCs) between layers of Matrigel or Geltrex hydrogel.^[^ ^7^, ^8^ ^]^ Recent advances have made use of microfluidic devices to seed human PSCs into pre‐formed Geltrex gel pockets and generated an asymmetric embryonic sac with epiblast and amniotic ectoderm components to recapitulate successive key events reflecting amnion cavity development in the post‐implantation stage.^[^ ^9^ ^]^

The extracellular matrix (ECM) interacting with individual cell types during human embryo development is critical for the epiblast layer formation and organogenesis.^[^ ^10^ ^]^ However, little is known about the detailed features of ECM in embryos and how they regulate early embryo development due to the scarcity of human embryo samples. Thus, the reported integrated and non‐integrated models are primarily generated in Matrigel or Geltrex, which are both extracted from mouse sarcomas and not well‐defined. There is an urgent need to design a well‐defined biomimicking matrix for in vitro embryo‐like models to support specific biological events. Moreover, fundamental research necessitates large quantities of reproducible models, which requires engineering methods with higher throughput than cell embedding and microfluidic devices. Bioprinting is a fast‐growing bioengineering method with advantages in mass production and 3D assembly of stem cells and ECM‐like hydrogel.^[^ ^11^, ^12^ ^]^ Various bioprinting strategies have been reported to efficiently generate tissue models using PSC‐derived cells or multipotent stem cells.^[^ ^13^, ^14^ ^]^ Our previous studies have reported microextrusion‐based bioprinting of PSCs and revealed cellular spheroids formation and pluripotency maintenance within the matrix.^[^ ^15^, ^16^, ^17^ ^]^ To our knowledge, bioprinting of embryo models with biomimicking disc‐like morphogenesis and gastrulation events was an untouched area.

**Figure 1:**
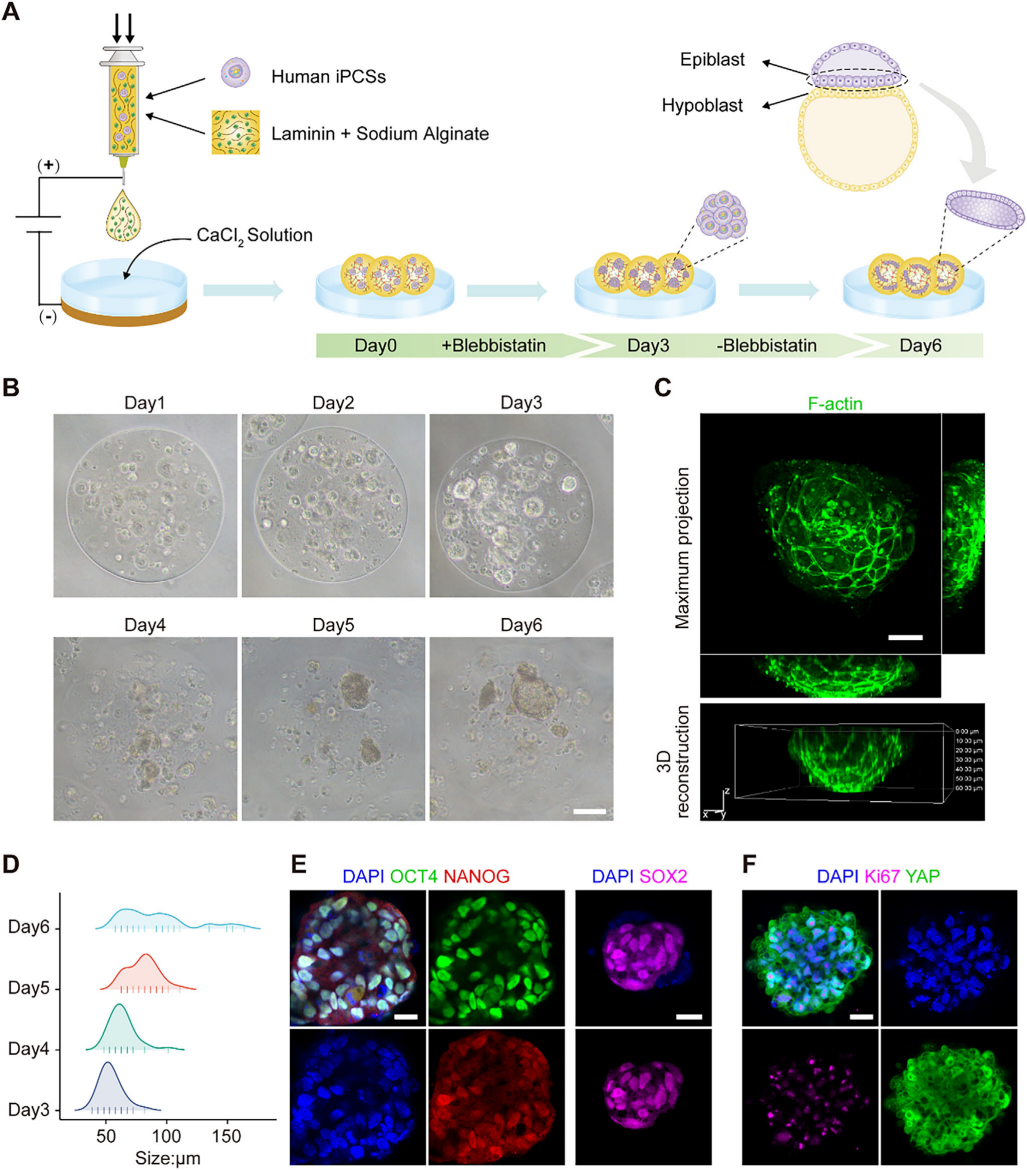
hiPSCs form epiblast‐like structures with disc morphology in the well‐defined bioink. A) Schematic presentation of the study design and flow. B) Optical microscope images of hiPSCs clusters within L‐A microgels showing temporal evolution of morphology. Scale bar, 100 µm. C) Immunofluorescence images of F‐actin in cell clusters on day6. The maximum projection of the orthogonal view is shown at the top with 50% Z zoom; the 3D reconstruction is shown at the bottom. Scale bar, 25 µm. D) Size distribution of cell clusters. E) Immunofluorescence staining images of OCT4, NANOG and SOX2 in disc‐like structures on day6. Scale bar, 25 µm. F) Immunofluorescence images of Ki67 and YAP in disc‐like structures on day6. Scale bar, 25 µm.

This study introduced a mild electro‐assisted bioprinting technique compatible with human induced pluripotent stem cells (hiPSCs) and human embryonic stem cells (hESCs). This method enables the mass production of micron‐sized microgels, which facilitates mass transfer owing to their porosity and large specific surface area.^[^ ^18^ ^]^ Furthermore, the spheroidal microgels exhibit structural characteristics that resemble those of blastocysts during the early stages of embryonic development. We proposed a laminin/alginate matrix (termed L‐A) to produce microgels for the generation of large quantities of non‐integrated human epiblast‐like models (**Figure** **1**A) since laminin is a ubiquitous basement membrane component and among the first detected during mammalian embryogenesis.^[^ ^19^, ^20^ ^]^ Unlike previous reports, this study utilized a well‐defined bioink and a self‐renewing medium without extra induction cytokines, providing a clean background decoupled from cytokine cues in the bioprinting niche. The specific niche facilitated efficient self‐organization of hiPSCs into epiblast identity and disc‐like morphogenesis and drove them toward gastrulation fate. Notably, the bioactive components and physical confinement provided by the L‐A bioink, and the endogenous regulation of the WNT signaling pathway, contribute to the disc‐shaped morphogenesis, recapitulation of epithelial‐to‐mesenchymal transition (EMT) patterns during gastrulation, and the generation of the posterior epiblast population. This study offers reproducible and manipulable epiblast‐like models reflecting gastrulation events in vitro and provides an exciting opportunity to investigate the effects of biochemical and biophysical cues on early embryo development, thereby deepening comprehension of birth defects, drug safety during pregnancy and regenerative medicine.

## 2 Results and Discussion

### 2.1 Electro‐Assisted Bioprinting of hiPSCs

Electro‐assisted bioprinting is a gentle process for generating large quantities of cell‐laden hydrogel microgels.^[^ ^11^, ^21^ ^]^ In our previous studies, this method was employed to bioprint human adipose‐derived stem cells,^[^ ^14^ ^]^ as well as hiPSC‐derived hepatic endoderm and endothelial progenitor cells,^[^ ^22^ ^]^ resulting in the generation of biomimetic organoids with specific genetic backgrounds. Furthermore, the principles of regulating printing parameters were systematically investigated to establish a stable and cell‐compatible bioprinting process.^[^ ^14^, ^22^ ^]^ This method is deemed appropriate for stem cells due to low viscosity and minimal shear stress, where a low concentration of polysaccharide sodium alginate is commonly used as the bioink's backbone component.^[^ ^11^, ^14^, ^23^ ^]^ But to our knowledge, electro‐assisted bioprinting has not been applied to hiPSCs or hESCs yet.

Here, we explored the bioprinting of hiPSCs by optimizing bioink's bioactive component. Considering that a uniform and sustainable bioprinting process is vital for mass‐generation of tissue models, we chose a subtype of laminin (laminin511 E8 fragments), the minimal functional form retaining the full capability of α6β1 integrin binding, ^[^ ^19^ ^]^ as the bioink's bioactive component, where Matrigel was used as a parallel control group. As for the commonly used Matrigel, we noticed the formation of floc‐like components upon mixing with sodium alginate, which randomly dispersed into Matrigel/alginate (termed M‐A) microgels (Figure S1A, Supporting Information). After culturing with the hiPSCs self‐renewing medium for 6 days, hiPSCs clusters with cavity structures and varying sizes (118.2 ± 40.7 µm) were obtained within the M‐A microgels, where cavity clusters were commonly observed among the floc‐containing regions (Figure S1B,C, Supporting Information), suggesting an inhomogeneous and unrepeatable microenvironment, adverse for large‐scale tissue model creation. On the other hand, laminin exhibited superior solubility when mixed with alginate, and few water‐insoluble matters were observed among bioprinting L‐A microgels (Figure S1A, Supporting Information). Bioprinting of L‐A bioink generated ≈6800 microgels per minute with uniform size of 360.5 ± 21.9 µm. Surprisingly, instead of cavity structures generated in M‐A bioink and previous reports, we observed uniform cellular self‐organization into disc‐like structures in L‐A bioink, with a constant upward‐facing opening orientation and gradually growing size in the bioink matrix, mimicking epiblast layer in early human embryos (Figure 1B–D; Figure S1C and Video S1, Supporting Information). We assumed the gravity force might play a role in the orientation of disc‐like structures since the microgels of this size range were spontaneously suspended in the culture medium. L‐A bioink also showed increased clustering efficiency (7.3 times) compared with M‐A bioink on day6 (Figure S1C,D, Supporting Information). Moreover, we found that hiPSCs cultured on 2D plates exhibited a shorter doubling time and a higher proliferation index compared to those cultured within 3D microgels (both L‐A and M‐A bioink) (Figure S2A,B, Supporting Information), indicating that the 3D microgel environment decelerate the proliferation rate of hiPSCs while promoting cellular self‐assembly. Our results revealed that self‐organization and morphogenetic events were critically regulated by the type of adhesive ligands presented in the bioink.

Next, we investigated the concentration of the backbone matrix, vital for optimal cell viability and microgel roundness. We set alginate concentrations at 2.5%, 3.5%, 4.5%, and 5.5% (w/v), respectively. We noticed similar shear‐thinning properties among different concentrations, beneficial for the flow of hiPSCs‐laden bioink during printing (Figure S3A, Supporting Information). After bioprinting, 3.5% sodium alginate showed optimal results for both microgel circularity (0.99 ± 0.001) (Figure S3B, Supporting Information) and cell survival (90.1 ± 0.8%) (Figure S3C, Supporting Information). Thus, the combination of 3.5% sodium alginate and 5 µg mL^−1^ laminin511E8F were determined as the bioink for hiPSC niche bioprinting. Given that viscoelastic characteristic is a common feature of nature tissues and ECMs,^[^ ^24^ ^]^ we performed rheological analysis and revealed the viscoelasticity of cross‐linked L‐A, with the storage modulus (G′’, indicating elasticity) at 5.7 kpa and the loss modulus (G″, indicating viscosity) at 1.4 kpa (Figure S3D, Supporting Information). These values were within the range of human soft tissues,^[^ ^25^ ^]^ suggesting the biomimetic mechanical property of L‐A niche.

As a key component of the bioink, the effect of initial hiPSC density on the printing process and cluster formation cannot be overlooked. To identify the optimal cell density for bioprinting hiPSCs and facilitating disc‐like morphogenesis, we explored a gradient of cell densities, including 1 × 10^6^ cells mL^−1^, 3 × 10^6^ cells mL^−1^, and 9 × 10^6^ cells mL^−1^. The bioinks with varying cell densities showed shear‐thinning properties (Figure S4A, Supporting Information). Compared to the initial density of 3 × 10^6^ cells mL^−1^, 1 × 10^6^ cells mL^−1^ exhibited comparable microgel circularity (0.99 ± 0.002) but lower clustering efficiency (≈8 clusters / mm^2^ microgels on day6) (Figure S4B, Supporting Information). In contrast, 9 × 10^6^ cells mL^−1^ displayed inferior morphology, characterized by lower microgel circularity (0.84 ± 0.073) and numerous broken microgels on day6 (Figure S4B, Supporting Information). This observation was consistent with the previous study demonstrating that high cell density interferes with bioink crosslinking, adversely affecting printed structure's shape fidelity and mechanical stability.^[^ ^26^ ^]^ Furthermore, we found that hiPSCs consistently self‐organized into disc‐like structures, regardless of the cell density in the bioinks (Figure 1C; Figure S4C, Supporting Information). These results indicated that the initial hiPSC density affected the printability of bioink, the mechanical properties of the printed structures, and the efficiency of cell clustering. An initial density of 3 × 10^6^ cells mL^−1^ was identified as the optimal option for subsequent investigations.

Taken together, an appropriate hiPSC niche could be bioprinted using a well‐defined bioink, thus directing the formation of large quantities of cellular clusters with biomimetic disc‐like morphogenesis.

### 2.2 Identity of Disc‐Like Structures in the Bioprinting Niche

Upon the emergence of the disc‐like morphology, we focused on analyzing the identity of these cellular structures. Pluripotency maintenance and continuous cell proliferation are crucial features of the epiblast population.^[^ ^27^ ^]^ Epiblast‐specific markers OCT4, NANOG, and SOX2 were positively expressed within the disc‐like tissues (Figure 1E), indicating their pluripotent potential. These structures also maintained proliferation with positive expression of Ki67 and nuclear localization of YAP (Figure 1F), consistent with the theory that nuclear activity of YAP promotes hPSCs proliferation,^[^ ^28^ ^]^ which fits the hallmark of the epiblast layer.

**Figure 2:**
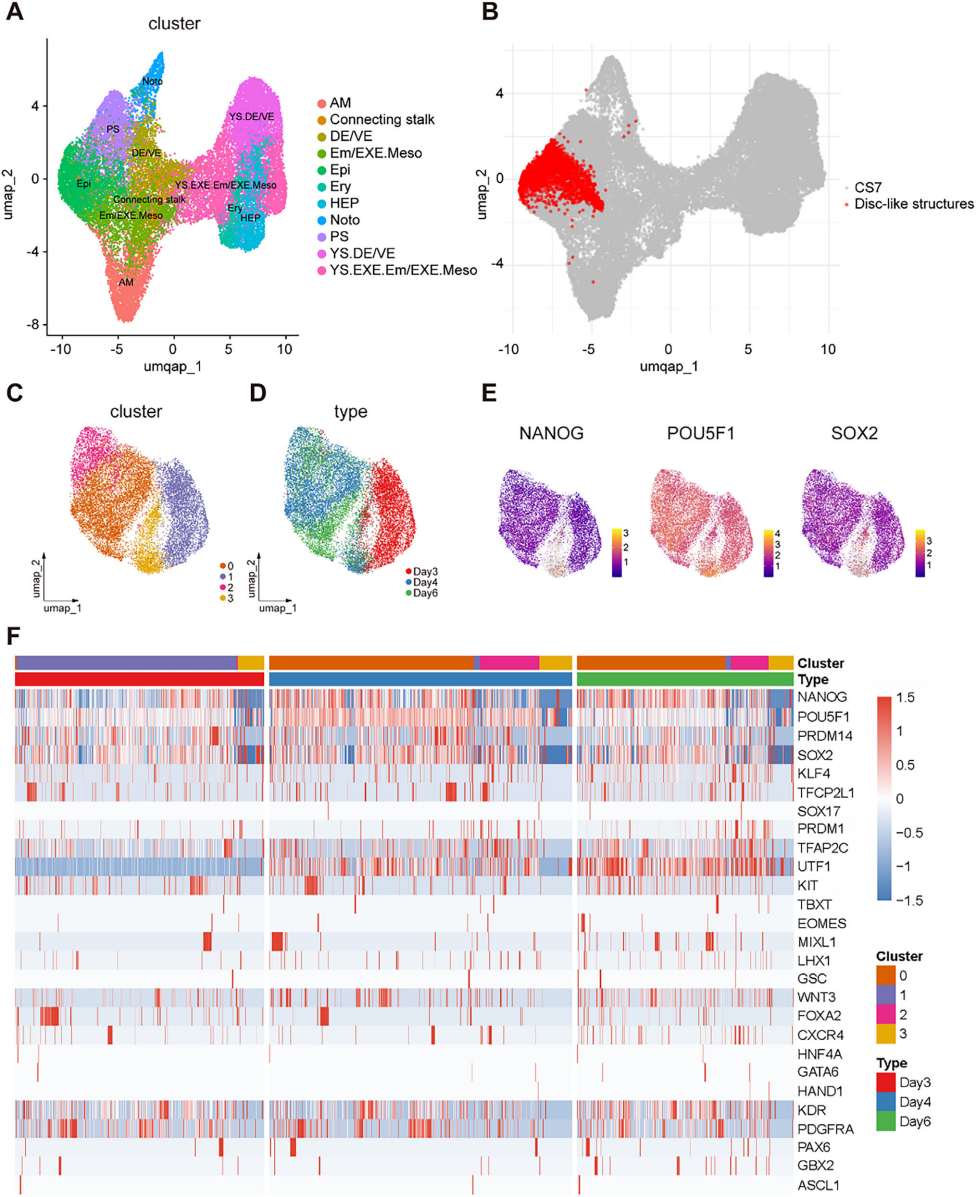
Single‐cell transcriptional profiling of disc‐like structures. A) UMAP of single‐cell transcriptomics of cells from the published dataset of the CS7.^[^ ^29^ ^]^ B) Individual cells from disc‐like structures are mapped to the CS7 human embryo reference coordinate system. C,D) UMAP of single‐cell transcriptomics of hiPSC clusters at different time points. Individual cells are colored based on classified clusters C) and their origin D). E) Gene expression of epiblast markers. F) A heatmap depicting the gene expression of specific lineage markers.

We then performed single‐cell RNA sequencing (scRNA‐seq) via the 10x Genomics platform to further determine the transcriptional composition of the disc‐like structures. Comparison with the published single‐cell transcriptomes of the Carnegie stage 7 (CS7) human embryo indicated that cells in the disc‐like tissues were transcriptionally similar with the epiblast lineage (**Figure** **2**A,B). Transcriptome profiling of disc‐like tissue derivation at different time points revealed that epiblast markers (NANOG, POU5F1, SOX2) expressing cells occupied almost the entire region in the uniform manifold approximation and projection (UMAP) space (Figure 2C–E). In contrast, other markers characterizing endoderm (SOX17, FOXA2, HNF4A), mesoderm (HAND1, KDR, PDGFRA), ectoderm (PAX6, GBX2, ASCL1), and primitive streak (TBXT, EOMES, MIXL1) exhibited low or nearly absent expression, indicating the epiblast identity without lineage segregation (Figure S5A–D, Supporting Information). Additionally, few changes to these lineage‐specific genes were observed at different time (Figure 2F). These results generally confirmed that disc‐like tissues within microgels faithfully mimicked the human epiblast layer.

### 2.3 Disc‐Like Structures Recapitulate Gastrulation‐Stage EMT Pattern

**Figure 3:**
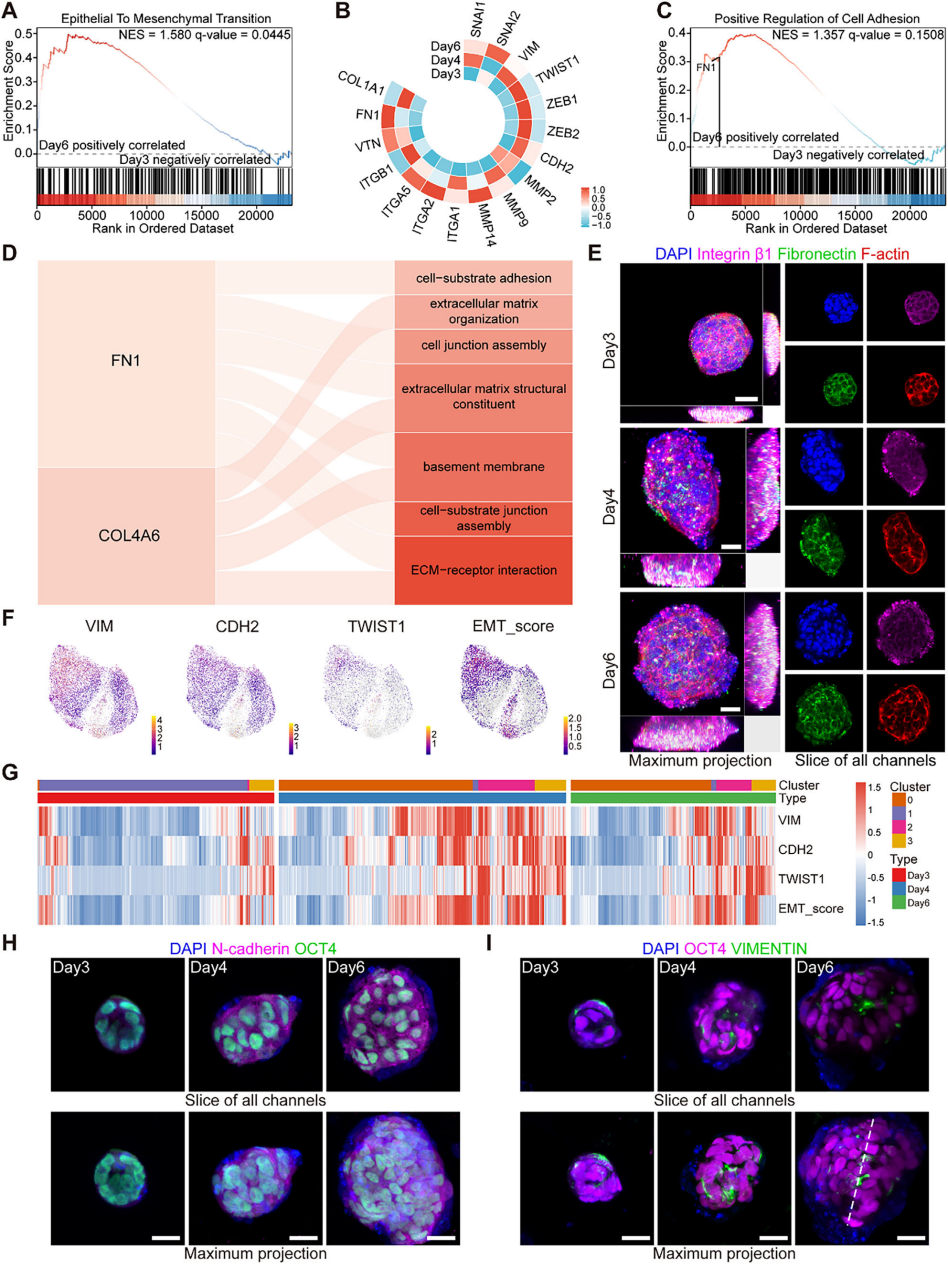
Characteristics of EMT during disc‐like structure formation. A) A representative plot of gene set enrichment analysis (GSEA) depicting EMT in the comparison of clusters on day6 versus day3. B) A heatmap of representative gene expression for EMT. C) A representative plot of GSEA depicting cell adhesion in the comparison of clusters on day6 versus day3. D) Sankey diagram indicating the key ECM components involved in cellular interaction with surroundings. E) Immunofluorescence co‐staining images of Fibronectin, Integrin β1 and F‐actin in epiblast‐like structures. Z zoom, 50%. Scale bar, 25 µm. F) Gene expression of EMT‐related markers. The EMT score is estimated by the expression of presented markers. G) A heatmap depicting the gene expression of EMT‐related markers. H, I) Immunofluorescence images of N‐cadherin, OCT4 H) and VIMENTIN, OCT4 I) in epiblast‐like structures within L‐A microgels. White dashed in I) denotes the axis crossing through the epiblast‐like cluster. Scale bar, 25 µm.

Human epiblast development is a spatiotemporally dynamic process, which can be divided into inner cell mass, pre‐implantation epiblast, post‐implantation epiblast, and primitive streak anlage‐epiblast.^[^ ^27^ ^]^ EMT in the specific region, indicative of primitive streak formation, is a critical event of gastrulation, which establishes the trilaminar disc of the body plan through spatiotemporally ordered morphological transformations.^[^ ^4^, ^30^ ^]^ Through bulk‐RNA sequencing (bulk RNA‐seq), we found upregulation of EMT at the transcriptome level after day3 (**Figure** **3**A,B). During EMT, the interactions of cell‐cell and cell‐ECM are remodeled,^[^ ^31^ ^]^ as exhibited in this study (Figure S6A,B, Supporting Information). FN1, an extracellular matrix protein and EMT regulatory molecule,^[^ ^32^ ^]^ was upregulated and involved in the regulation of cell adhesion after day3 (Figure 3B–D). At the protein level, clusters within L‐A microgels maintained expression of fibronectin and binding integrin subunit integrin β1 (Figure 3E). Since no exogenous fibronectin was introduced into the bioink system, these self‐organized cell clusters showed the capacity to secrete fibronectin autonomously. Next, scRNA‐seq analyses delineated the heterogeneity of EMT within disc‐like tissues, demonstrating higher expression levels of EMT‐specific genes (VIM, TWIST1, and CDH2) and EMT score in the classified cluster 2 predominantly comprised cells on day4 and day6 (Figure 3F,G). Immunostaining of N‐cadherin showed increasing accumulation at the cell junction on day4 and day6 (Figure 3H). VEMINTIN, a typical mesenchymal marker, also exhibited increasing expression and tended to localize in the region near the axis crossing through the center of epiblast‐like tissues after day3 (Figure 3I), which was reminiscent of the EMT occurring at the primitive streak structure situated along the midline of the gastrulating embryo.^[^ ^6^ ^]^ These findings suggested that the interactions between cells and their surrounding environment undergo remodeling during the formation of disc‐like structures, along with the appearance of an EMT pattern recapitulating the gastrulation stage.

### 2.4 Disc‐Like Structures Mimic the Posterior Epiblast Population

**Figure 4:**
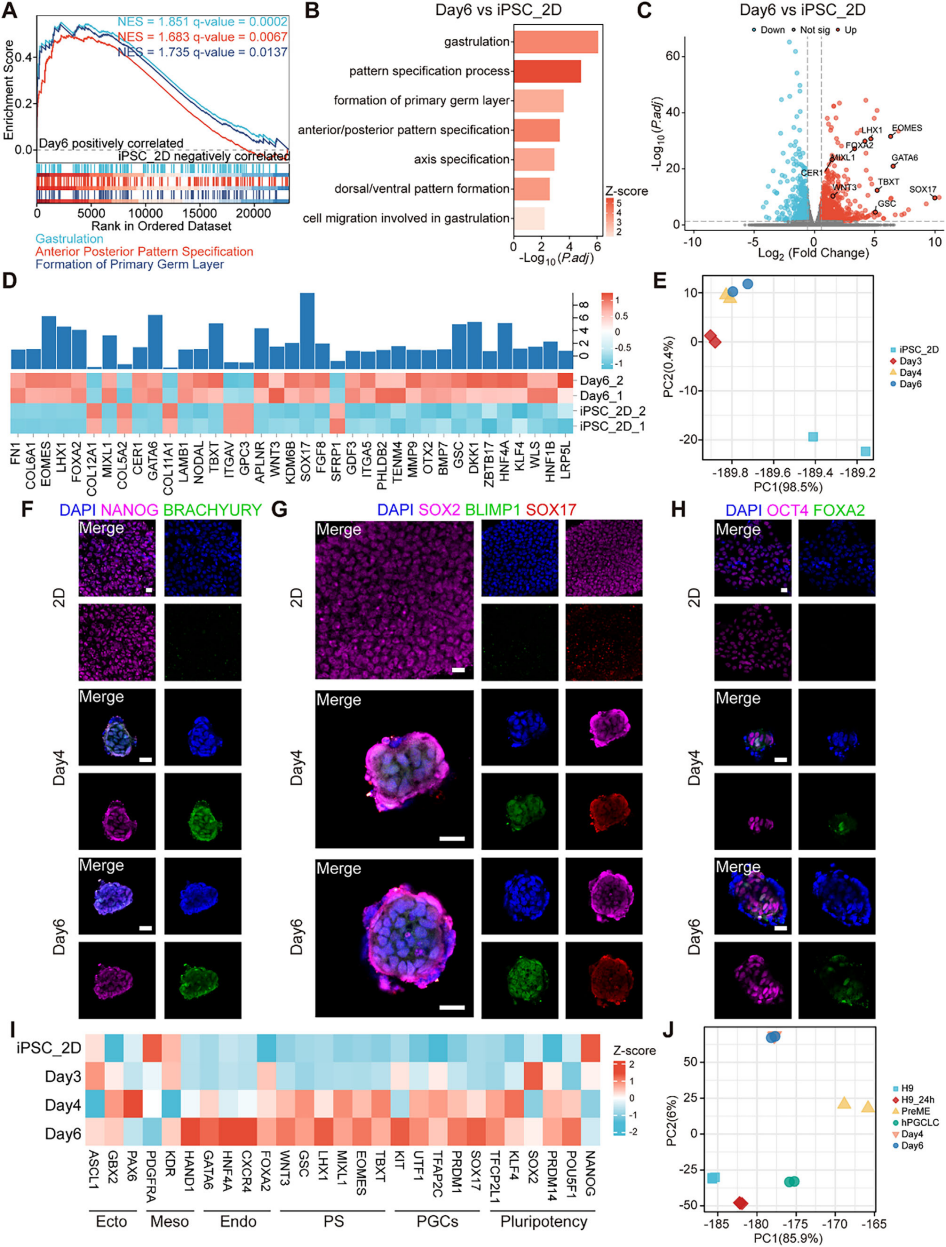
Disc‐like structures featuring a gastrulation fate resembling the posterior epiblast population. A‐C) Representative plots of GSEA A), column chart depicting gene ontology (GO) enrichment B) and volcano map depicting differentially expressed genes (DEGs) C) for the comparison of samples on day6 versus iPSC_2D. D) A heatmap illustrating gene expression associated with the gastrulation term in GO enrichment. E) Principal component analysis (PCA) of RNA‐seq data for samples from 2D cell culture plates and L‐A microgels at indicated time points. F‐H) Immunofluorescence co‐staining images of NANOG, BRACHYURY F), SOX2, BLIMP, SOX17 G) and OCT4, FOXA2 H) in 2D cultured hiPSCs and disc‐like structures. In the control group, 2D hiPSCs are cultured on laminin511E8F‐coated 12‐well plates. Scale bar, 25 µm. I) A heatmap of representative gene expression for pluripotency, primordial germ cells (PGCs), primitive streak (PS), endoderm (Endo), mesoderm (Meso), and ectoderm (Ecto). J) PCA of RNA‐seq data for samples from L‐A microgels on day4 and day6 alongside published datasets.^[^ ^36^, ^37^ ^]^

To delve deeper into the corresponding developmental status of these disc‐like structures, we analyzed the transcriptome of cell clusters throughout the whole culture period. Cell clusters on day6 exhibited a tendency for gastrulation compared to hiPSCs grown in 2D cell culture plates in the light of enrichment analysis of differentially expressed genes (**Figure** **4**A,B; Figure S7A, Supporting Information) and the upregulation of gastrulation‐related genes (Figure 4C,D; Figure S7B, Supporting Information). Furthermore, cell clusters on day4 and day6 displayed a closer transcriptional resemblance, suggesting their proximity in identity (Figure 4E). We regarded the day3 as a turning point, after which the clusters underwent gastrulation‐related specification (Figure S7C–H, Supporting Information). Subsequently, immunostaining analysis with specific lineage markers revealed hiPSCs grown in 2D cell culture plates exclusively expressed pluripotency markers (NANOG, SOX2, OCT4) (Figure 4F–H). In contrast, clusters on day4 and day6 co‐expressed markers characteristic of pluripotency, primitive streak cells (BRACHYURY), and human primordial germ cells (SOX17, BLIMP1), along with minor expression of endoderm‐specific markers (FOXA2) (Figure 4F–H), indicating their possible status equivalent to mesendoderm precursors (PreME).^[^ ^33^ ^]^ Increased expression of genes associated with specific lineages was also observed in the clusters on day4 and day6 (Figure 4I). Compared to reference datasets, we found that clusters on day4 and day6 were transcriptionally most closely correlated with PreME (Figure 4J). Our results demonstrated that the 3D L‐A microgel microenvironment enhanced the formation of disc‐like tissues with a specific identity corresponding to the posterior epiblast population of human pre‐gastrulation embryos,^[^ ^33^ ^]^ with the competency differentiating into endoderm and mesoderm under cytokine induction (Figure S8A,B, Supporting Information).

Notably, the myosin II inhibitor blebbistatin, essential for rescuing PSCs from dissociation‐induced apoptosis,^[^ ^34^ ^]^ was removed on day3 coinciding with the observation of cell aggregation and a shift in the fate of cell clusters. This prompted the assumption that removing blebbistatin initiates gastrulation‐related specification of the cell clusters encapsulated within microgels. To further elucidate the role of blebbistatin, we conducted experiments in which blebbistatin was either absent or present throughout the entire culture period. Without blebbistatin, hiPSCs within microgels failed to self‐organize into disc‐like structures (Figure S9A, Supporting Information). Conversely, when blebbistatin was continuously present, we observed the emergence of cavity clusters (Figure S9A,B, Supporting Information). Neither the complete absence nor the persistent presence of blebbistatin was conducive to the formation of disc‐like structures. Besides, transcriptomic and immunostaining analyses revealed that the persistent presence of blebbistatin inhibited cell lineage specialization to the gastrulation (Figure S9C–F, Supporting Information), consistent with the previous study confirming that inhibition of myosin II can block lineage specification.^[^ ^35^ ^]^ These results indicated that timely addition and removal of blebbistatin facilitated the generation of disc‐like posterior epiblast models with a propensity for gastrulation within microgels.

We then attempted to produce disc‐like tissues in printed L‐A microgels using human ESCs. Similar to hiPSCs, the encapsulated ESCs exhibited a high level of viability (Figure S10A, Supporting Information) and self‐organized into disc‐like structures (Figure S10B, Supporting Information) resembling the posterior epiblast population (Figure S10C–E, Supporting Information), indicating that this technology is robust and can be successfully replicated among PSCs. These results were consistently reproduced using an alternative self‐renewing medium (Figure S11, Supporting Information). Together, we have developed a robust and versatile strategy for generating large‐scale disc‐like epiblast models in vitro using a well‐defined bioink and a self‐renewing medium.

### 2.5 Physical Confinement and Bioactive Component of 3D Biomatrix Regulate the Morphogenesis of Disc‐Like Tissues

**Figure 5:**
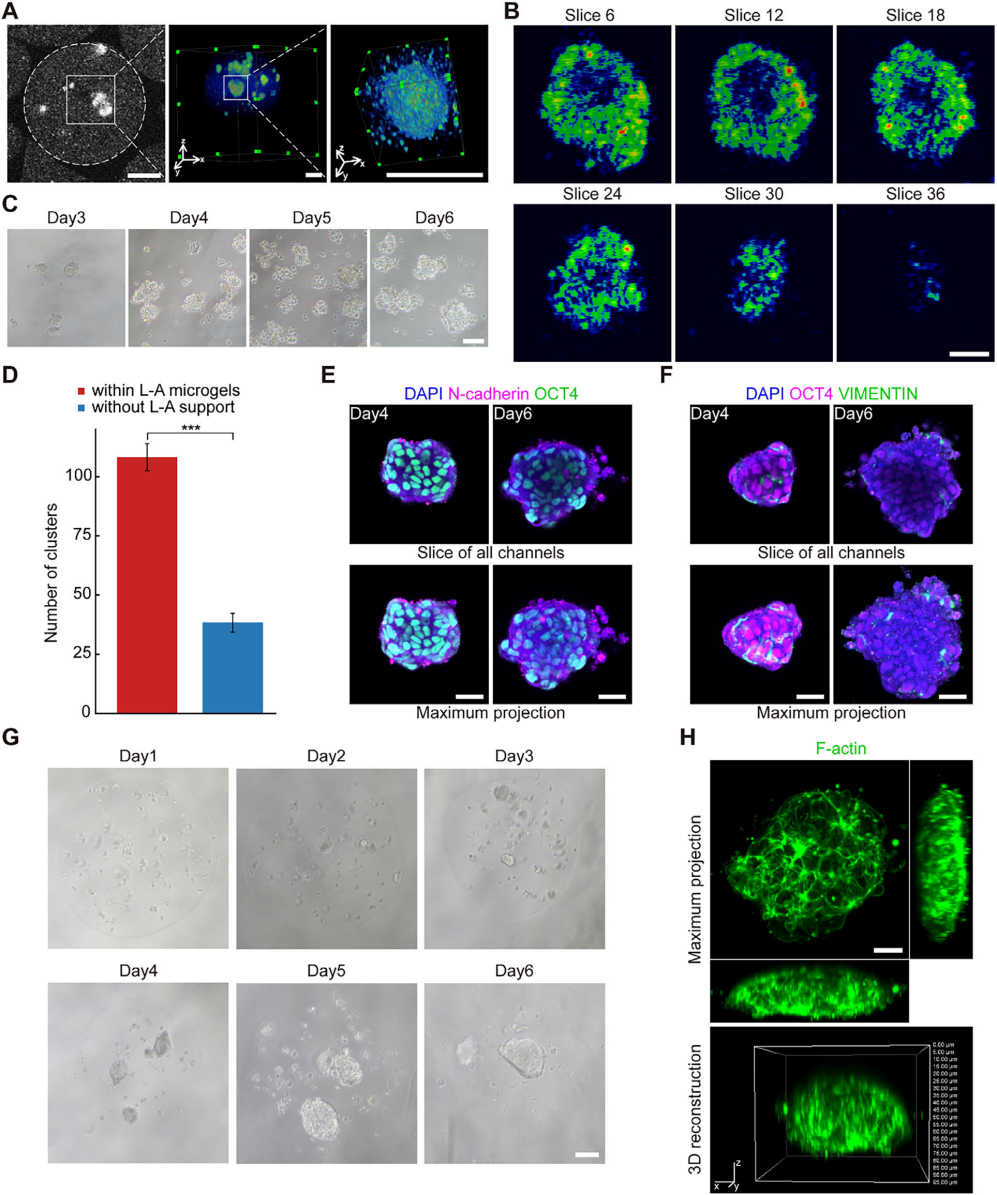
Biomatrix regulates gastrulation‐stage morphogenesis of disc‐like tissues. A) OCT imaging for the disc‐like structure wrapped in L‐A microgels (day6). Images include en face projection (left), 3D rendering (middle) and the magnifying tissue of 3D rendering (right). Blue in 3D rendering represents the biomatrix (L‐A), and green represents cells. Scale bar, 100 µm. B) OCT imaging slices of the disc‐like structure (day6). Scale bar, 25 µm. C) Optical microscope images showing cluster morphology after removal of L‐A biomatrix. Scale bar, 100 µm. D) Comparison of cluster generation with and without L‐A hydrogel support. The vertical axis denotes the number of clusters per well of a 12‐well plate on day6. Data are presented as mean ± s.d. (n = 6 biological replicates). Data are statistically analyzed using independent‐samples T test. *** means P < 0.001. E, F) Immunofluorescence co‐staining images of N‐cadherin, OCT4 E) and VIMENTIN, OCT4 F) in clusters without L‐A support. G) Optical microscope images of hiPSCs clusters within pure alginate microgels. Scale bar, 100 µm. H) Immunofluorescence images of F‐actin in clusters within pure alginate microgels on day6. The maximum projection of the orthogonal view is shown at the top with 50% Z zoom; the 3D reconstruction is shown at the bottom. Scale bar, 25 µm.

Regional cell extrusion and delamination under the local mechanical stimulation promote processes of EMT, ingression and well‐ordered cell packing.^[^ ^38^, ^39^, ^40^ ^]^ Additionally, physical compression is confirmed to cause heterogeneous subpopulations with epithelial or mesenchymal features.^[^ ^41^ ^]^ Employing optical coherence tomography (OCT) imaging, it was observed that disc‐like tissues were wrapped among the cross‐linked L‐A microgels (**Figure** **5**A,B; Videos S2 and S3, Supporting Information). The diverse intrinsic backscattering characteristics exhibited by disc‐like tissues, culture media, and L‐A biomatrix enabled OCT to visualize the complex 3D morphology of disc‐like tissues within the microgels. Previous understanding indicated that ECM regulates morphological properties of tissues through biomechanical cues.^[^ ^42^ ^]^ Thus, we hypothesized that the gastrulation‐stage EMT pattern of disc‐like structures potentially arose from physical compression induced by spatial confinement of 3D biomatrix. To validate this hypothesis, cell clusters were collected from hydrolyzed L‐A microgels on day3, and subsequently resuspended in the self‐renewing medium for continued cultivation. Numerous fragmentary tissues were observed in the absence of L‐A hydrogel support (Figure 5C), resulting in a significantly reduced cluster generation (Figure 5D). Despite only a few clusters growing without the support of biomatrix, we investigated the cellular behavior exhibited by these remaining clusters. Similar to the samples within L‐A bioink, the remaining clusters contain tight junction protein ZO‐1 secreting cells localizing at the outer edge of cell clusters (Figure S12, Supporting Information). We then noticed a comparable expression of fibronectin but a decreased expression of integrin β1 in the remaining clusters (Figure S13, Supporting Information). We assumed the formation of tight junctions and the secretion of ECM components contributed to the maintenance of structural integrity of the cell clusters without the support of biomatrix. Subsequently, we assessed the expression of EMT‐specific markers in the remaining clusters. Immunofluorescence analyses revealed minimal accumulation of N‐cadherin at the cell junction (Figure 5E) and a scattered expression of VEMINTIN lacking the typical spatial features (Figure 5F), suggesting hampered spatiotemporal characteristics associated with gastrulation‐stage EMT. The findings indicated that the physical confinement of the L‐A biomatrix contribute the structural integrity of cell clusters and the spatial pattern of EMT.

Besides physical confinement, the biomatrix also offers biochemical cues. This study proposed using a well‐defined printing bioink, allowing for the investigation of the effects of bioactive components on cellular behaviors. Upon the exclusion of laminin511E8F from the printing bioink and generating pure alginate microgels containing hiPSCs, we observed that hiPSCs self‐organized into spheroidal structures within the alginate microgels rather than disc‐like morphology (Figure 5G,H; Video S4, Supporting Information), suggesting that exogenous adhesion sites provided by laminin guided the specific morphogenesis of hiPSC clusters.

Together, these results demonstrated that the physical confinement provided by the 3D biomatrix was crucial for maintaining the clustering efficiency and gastrulation‐related EMT pattern in the epiblast‐like models. Additionally, the presence of the exogenous bioactive component (laminin511E8F in this study) within the 3D biomatrix played a key role in directing disc‐like morphogenesis.

### 2.6 WNT Signaling Pathway Regulates Gastrulation Fate of Disc‐Like Tissues

**Figure 6:**
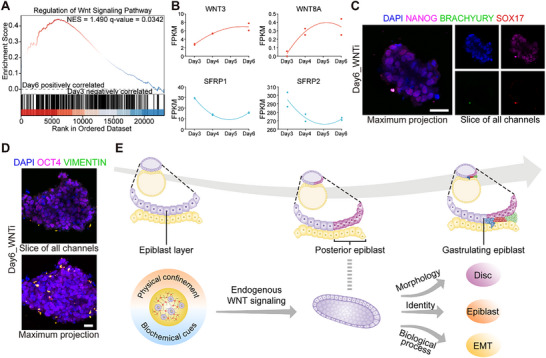
WNT signaling pathway regulates the gastrulation‐stage characteristics of disc‐like structures. A) A representative plot of GSEA depicting WNT signaling pathway in the comparison of clusters on day6 versus day3. B) Dynamic expression wave of WNT signaling pathway related genes. C,D) Immunofluorescence co‐staining images of NANOG, BRACHYURY, SOX17 C) and VIMENTIN, OCT4 D) in clusters on day6 with WNT inhibitor. E) The developmental process of the epiblast layer in vivo and the evolution of the posterior epiblast tissues from hPSCs in vitro.

The organization of signaling is vital for establishing the embryonic developmental axis, illuminating the pathway for the remarkable journey of pattern formation. As a crucial member of signaling pathways, WNT signaling pathway is essential to induce the formation of a primitive streak, which is defined as the onset of gastrulation.^[^ ^43^ ^]^ Upon evaluating bulk RNA‐seq, we noticed that the regulation of WNT signaling pathway contributed to the shifts in gene clusters between day6 and day3 (**Figure** **6**A). Additionally, there was an upregulated expression of WNT ligands (WNT3, WNT8A) and downregulated expression of WNT inhibitors (SFRP1, SFRP2) (Figure 6B). To verify the role of the WNT signaling pathway in the epiblast‐like models, we applied a small‐molecule inhibitor of the WNT signaling pathway starting on day3. Upon treatment, cell clusters on day6 exhibited positive expression of NANOG, whereas BRACHYURY and SOX17 were not expressed (Figure 6C), suggesting WNT signaling pathway contribute to the cell fate decision. Furthermore, the expression of VIMENTIN was nearly undetectable (Figure 6D). These results substantiated the role of the endogenous WNT signaling pathway in regulating gastrulation‐stage characteristics of self‐organized hiPSC clusters within the L‐A microgel microenvironment. In summary, the endogenous regulation of the WNT signaling pathway, combined with physical and biochemical cues from the 3D microgels, could collectively facilitate the generation of in vitro models mimicking the posterior epiblast population (Figure 6E).

## 3 Conclusion

We have developed a versatile strategy, the mild electro‐assisted bioprinting technology, to effectively generate disc‐like epiblast tissues that closely resemble the posterior epiblast population of human embryos around the gastrulation stage. In this study, a well‐defined bioink comprising bioactive and backbone components was used to promote the construction of a biomimetic niche. Laminin, serving as the bioactive component, directed the disc‐shaped morphogenesis. The physical confinement provided by the 3D biomatrix contributed to the model generation efficiency and gastrulation‐like EMT pattern, holding great promise to construct primitive streak‐like structures in vitro. The design of a well‐defined bioink not only facilitates the controllable production of human epiblast models with a homogeneous identity but also enables the investigation of the mechanisms by which various ECM components regulate morphogenesis, which aims to address a gap in the understanding of how the specific characteristics of the ECM influence early embryonic development.

Furthermore, the existing early embryonic models remain difficult to faithfully replicate longer‐term developmental processes due to uncontrolled collapse and unsustainable lineage differentiation.^[^ ^3^, ^9^, ^44^ ^]^ This work confirms that introducing an appropriate biomatrix is expected to overcome the technical limitations associated with the long‐term culture of artificial embryo models in vitro caused by structural collapses and supports the growth of embryo models for later stages of embryogenesis.

This study represents the first attempt to generate epiblast models utilizing bioprinting technology. A self‐renewing medium is employed throughout the process, thereby mirroring the functionality of 3D microenvironment constructed from a well‐defined bioink. However, the currently constructed microenvironment cannot fully replicate the uterine environment or model extraembryonic structures. Future research may integrate biochemical signals, including a broader array of cell types and cell‐inducing culture medium, to create a more biomimetic microenvironment that facilitates the generation of more sophisticated human embryo models capable of long‐term culture, thereby supporting further investigations into congenital disabilities, drug safety during pregnancy, and the field of regenerative medicine.

## 4 Experimental Section

### Cell Culture

Both hiPSCs and H9 ESCs were provided by Shownin Company and grown on Matrigel‐coated 6‐well plates using the commercial serum‐free self‐renewing medium (Shownin, RP01020) at 37 °C in a humidified incubator with 5% CO_2_. The medium was changed daily, and cells were passaged when reaching 80% confluency. In this study, hiPSCs at passages 25 to 35 and hESCs at passages 45 to 50 were used for the experiments.

### Preparation of Bioink

The sodium alginate powder (Sigma, A0682) was dissolved in a saline solution to prepare a 4% (w/v) sodium alginate solution. Subsequently, the sodium alginate solution was heated at 80 °C for 3 h. Following this, the solution was stirred on a magnetic stirrer (DLAB, MS‐H340‐S4) at a temperature of 37 °C and a stirring speed of 750 rpm for 1 h. This heating and stirring process was repeated three times, with the final stirring conducted overnight. The resultant material was then filtered using a 0.45 µm filter membrane to yield a 4% sodium alginate stock solution.

### Bioprinting for L‐A Microgels Laden hiPSCs

Human iPSCs were pre‐incubated for 1 h with 2.5 µM blebbistatin (Shownin, RP01008) and subsequently dissociated into single cells using Solase (Shownin, RP01021). A 4% sodium alginate solution, laminin511EBF/Matrigel and hiPSCs‐contained medium were mixed with the final cell density being 3 × 10^6^ cells mL^−1^. In comparing bioactive components, Matrigel (Corning, 354 277) was diluted 30‐fold, while laminin511E8F (Shownin, RP01025‐1) was diluted to a concentration of 5 µg mL^−1^. Next, we developed the electro‐assisted cell printing system in accordance with prior research.^[^ ^14^ ^]^ The system primarily comprised three components: a high‐voltage power (BMEI, BGG40/2), a syringe pump (Longer Pump Ltd) and a collection device. Microgels were produced using printing parameters of 12 kv voltage and a 10 ml h^−1^ push speed, and were subsequently cross‐linked with the CaCl_2_ solution.

### Collection and Culture for L‐A Microgels Laden hiPSCs

Crosslinked microgels were transferred to 50 ml centrifuge tubes. Following the deposition of the microgels at the bottom of the tubes, the CaCl_2_ solution was removed, and the microgels were washed three times. Subsequently, the microgels were resuspended in self‐renewing medium and evenly distributed into 12‐well plates, with 1.5 ml of medium in each well. The medium was changed daily. In particular, we replaced the medium with an alternative commercial serum‐free self‐renewing medium (STEMCELL, 85 850) for the repeated experiment. After three days of culture in medium containing 2.5 µM blebbistatin, the blebbistatin was removed, and the microgels were cultured for an additional three days. In the experimental investigation of the regulatory effects of the WNT signaling pathway, 2.5 µM WNT inhibitor (MedChemExpress, HY‐12238) was incorporated into the self‐renewing medium from day 3 to day 6. In the experiments involving proliferation rate assay, cluster counting, RNA sequencing and immunofluorescence staining, hiPSC clusters were obtained by hydrolyzing the microgels with a hydrolysate solution (containing 55 mM sodium citrate, 20 mM EDTA and 150 mM sodium chloride), followed by centrifugation at 50 g for 3 min.

### Proliferation Rate Assay

Around 5 × 10^4^ hiPSCs per well were seeded onto Matrigel‐ or Laminin511E8F‐ coated 12‐well plates to evaluate the proliferation rate under 2D culture conditions. Consistent with the hiPSC culture protocol used within microgels, cells were initially cultured for three days in a commercial serum‐free self‐renewing medium containing 2.5 µM blebbistatin, followed by an additional three days of culture after the removal of blebbistatin. At the designated time point, the number of dissociated single cells in each well was counted. To assess the proliferation rate within microgels, hiPSCs were dissociated into single cells following collection from hydrolyzed L‐A or M‐A microgels and subsequently quantified. The proliferation rate was characterized using the proliferation index (PI) and doubling time (DT), which were calculated according to the formulas: PI = (FD‐ID) / ID and DT = Time Duration × ln (2) / ln (FD / ID), where FD refers to the final cell density and ID refers to the initial cell density.

### Rheological Test

Rheological measurements were performed using a rotational rheometer (MCR302, Anton Paar) equipped with a 25 mm diameter plane‐plate. In viscosity curve mode, the viscosity of uncross‐linked L‐A was measured over a shear rate scan ranging from 0.1 to 1000 s^−1^. In oscillatory mode, the G′ and G″ of cross‐linked L‐A were recorded as functions of time, with a strain of 0.1% and a frequency of 1 Hz. The values of G′ (or G″) were determined as the average of all time points following the attainment of a plateau, and three samples were tested to calculate the mean values.

### Differentiation of Endoderm and Mesoderm

The differentiation protocol referred to the previous study.^[^ ^45^ ^]^ Here, the initiation of differentiation as Day 0 was designated. On day0, RPMI 1640 (Gibco, C11875500BT) containing 1 × B27 minus insulin (Gibco, A1895601), 100 ng mL^−1^ Activin A (R&D, 338‐AC) and 20 ng mL^−1^ BMP4 (PeproTech, AF‐120‐05ET) was used to culture epiblast‐like tissues for 5 h. And then the above medium supplemented with 25% self‐renewing medium was used for 2 days. Next, the medium was replaced with RPMI 1640 containing 1 × B27 minus insulin, 100 ng mL^−1^ Activin A and 25% self‐renewing medium for the following 2 days. The medium was changed daily.

### Immunofluorescence Staining

Immunofluorescence staining of clusters was according to a previously published protocol.^[^ ^46^ ^]^ All samples were fixed with a paraformaldehyde solution (Solarbio, P1112) at room temperature for 30 min. The fixation process was terminated with a buffer solution containing 0.1% Tween (Solarbio, T8220, diluted at a rate of 10:1) at 4 °C for 10 min. Following centrifugation at 50 g for 3 min, the supernatant was removed. Samples were then permeabilized with a buffer solution comprising 0.1% Triton (Beyotime, P0096) and 0.2% BSA (Yeasen, 36101ES60) at 4 °C for at least 1 h. Primary antibodies were added and incubated overnight at 4 °C in the dark. The dilutions of primary antibodies used were: rabbit anti‐OCT4 (1: 100; CST, 2750S), mouse anti‐OCT4 (1: 100; CST, 75463S), mouse anti‐NANOG (1:200; CST, 4893S), goat anti‐SOX17 (20 µg mL^−1^; R&D, AF1924), mouse anti‐SOX17 (20 µg mL^−1^; R&D, MAB19241), rabbit anti‐BRACHYURY (1:200; CST, 81694S), rabbit anti‐BLIMP1 (1: 200; abcam, ab198287), rabbit anti‐FOXA2 (1:200; CST, 8186S), mouse anti‐N‐Cadherin (1: 200; CST, 14215S), rabbit anti‐VIMENTIN (1:100; CST, 5741S), rabbit anti‐ZO1 (1:100; CST, 13663S), rabbit anti‐Ezrin (1:50; abcam, ab270442), rabbit anti‐YAP (1:100; CST, 14074S), mouse anti‐Ki67 (1:1000; CST, 9449S), rabbit anti‐Fibronectin (1:100; CST, 26836S), mouse anti‐Integrin β1 (1:100; abcam, ab30394) and F‐actin (1:500; abcam, ab176753 (iFluor‐488) / ab176757(iFluor‐594)). Next, samples were washed three times, with the initial two washes lasting 3 min each, followed by a third wash lasting 2 h. Hoechst (Solarbio, C0020) and secondary antibodies, including anti‐mouse (CST, 4410S), anti‐rabbit (CST, 4412S) and anti‐goat (abcam, ab150130), were added and incubated overnight at 4 °C in the dark. All secondary antibodies were diluted to a ratio of 1:200, while the working concentration of Hoechst was maintained at 10 µg mL^−1^. Finally, all samples were washed three times, with the first two washes lasting for 3 min each, followed by the third wash for 2 h. Following the immunofluorescence staining procedure, clusters were transferred to specialized confocal dishes for imaging. Images were acquired using a NIKON A1R HD25 confocal microscope that was equipped with a 40× silicone oil immersion objective and 20× objective. Image J software was used for image processing and quantification analysis of fluorescence.

### OCT Imaging

OCT, with its advantages of label‐free, non‐invasive, and non‐destructive imaging,^[^ ^47^, ^48^ ^]^ is particularly well‐suited for in‐situ, 3D imaging of tissues or organs within microgels without compromising subsequent culture or other detection procedures. High‐resolution spectral domain OCT, with a central wavelength of 880 nm, was utilized for 3D morphology data acquisition. The axial and lateral resolutions of the OCT system were 3.0 µm (in air) and 4.0 µm, respectively. The imaging field of view can be adjusted according to the imaging target, with a maximum size of 6*6 mm^2^. The imaging depth achieved was 1.9 mm in air, and the A‐Scan frequency was configured to 100 kHz. The system sensitivity was measured at ≈102 dB.

### Bulk RNA‐seq

The processes of RNA extraction, library preparation, quality control, library construction, and initial analysis for RNA sequencing were conducted by Novogene. RNA sequencing of the samples was performed using Illumina NovaSeq 6000, which generated 150 bp paired‐end reads. The reference genome index was built using HISAT2 (v2.0.5), and HISAT2 (v2.0.5) was used to align the paired‐end clean reads to the reference genome. The number of reads mapped to each gene was counted by featureCounts (1.5.0‐p3). Subsequently, the FPKM for each gene was calculated based on the gene length and reads count mapped to that gene. DESeq2 R package (1.20.0) was used to calculate DEGs with fold change ≥ 1.5 and padj<0.05. GO and kyoto encyclopedia of genes and genomes (KEGG) enrichment analysis for DEGs were performed using clusterProfiler R package with z‐score values being calculated via the GOplot R package (positive z‐scores indicated potential positive regulation, while negative z‐scores indicated potential negative regulation; larger absolute values of z‐scores reflected a higher degree of regulation). Additionally, we performed GSEA analysis on the GO and KEGG datasets using a local version of the GSEA analysis tool: http://www.broadinstitute.org/gsea/index.jsp.

### scRNA‐seq

The survival rate of isolated single cells exceeded 80%. Single‐cell GEMs were rapidly generated using the fully automated Chromium Controller, followed by reverse transcription into cDNA. The amplified cDNA was fragmented, end‐repaired, and A‐tailed, and then ligated with adapters. The resultant product was PCR‐amplified and circularized single‐stranded DNA molecules were produced with uncyclized linear DNA molecules being digested. These single‐stranded circular DNA molecules were replicated through rolling circle amplification to generate DNA nanoballs (DNBs) for sequencing. Sequencing and library construction were performed on the 10x Genomics platform (BGI).

### Single‐Cell Data Processing, Quality Control and Visualization

The single‐cell data were processed using the Cell Ranger pipeline (v.7.1.0) with default parameters. The reference and gene annotations were sourced from the 10X Genomics website (GRCh38‐2020‐A). Further analysis was performed in R (v.4.2.2) using Seurat (v.4.9.9).^[^ ^49^ ^]^ For each sample, genes that were not expressed in at least three cells and cells containing fewer than 200 genes were filtered out. Following the direct merging of the three samples, additional filtering was applied to remove cells with fewer than 500 detected genes, more than 12 000 detected genes and exceeding 20% mitochondrial gene percentage. Ultimately, a total of 14 623 cells were retained for downstream analysis. The raw count matrix was log‐normalized and scaled using the default method in Seurat. A selection of 2000 highly variable genes was used for PCA. The first 20 PCs were selected for UMAP visualization. The cells were then clustered using the Louvain algorithm^[^ ^50^ ^]^ with a resolution of 0.2. All procedures were performed using the default parameters of the Seurat functions RunPCA, RunUMAP, FindNeighbors, and FindClusters.

### Scoring and Density Plot

The AddModuleScore^[^ ^51^ ^]^ function in Seurat was used to generate the corresponding score. To enhance the visual presentation of these feature plots, scCustomize (v.1.1.1) was used. Furthermore, we employed kernel density estimation to create a density plot for the genes VIM, CDH2, and TWIST1.^[^ ^52^ ^]^ The expression levels of these three genes and the EMT score were represented as a heat map using the heatmap (v.1.0.12).

### Statistical Analysis

All data were presented as mean±s.d. Shapiro‐Wilk test for normal distribution was performed before significance analysis. Statistical significance was assessed by independent‐samples T test for two groups and one‐way ANOVA (Bonferroni multiple comparisons test) for three or more groups. Sample size was no less than 3 for each statistical analysis. Statistical difference was defined as P < 0.05. Statistical testing was analyzed using SPSS software. Graphs were generated using OriginLab or GraphPad Prism software.

## Conflict of Interest

The authors declare no conflict of interest.

## Author Contributions

R.Y., M.X. and X.W. conceptualized and designed all projects. Y.L. contributed key ideas, designed and carried out all experiments, and analyzed data. Y.L., L.L., L.W., S.Y. and H.R. wrote the manuscript. L.L., X.W. and H.R. performed scRNA‐seq analysis. L.W. and S.Y. performed OCT detection. S.L., X.W. and Y.S. participated in the bioprinting experiment. L.Y. contributed technical and scientific expertise to the manuscript. R.Y., L.Y., M.X. and X.W. revised and improved the manuscript. M.X., R.Y. and X.W. supervised the project.

## Supporting information



Supporting Information

Supplemental Video 1

Supplemental Video 2

Supplemental Video 3

Supplemental Video 4

## Data Availability

The data supporting the results in this study are available within the paper and its Supporting Information. Bulk RNA‐seq data are available at NCBI Gene Expression Omnibus under accession code GSE243362. Single cell RNA‐seq data are available at Genome Sequence Archive under accession code HRA008773. Published datasets used in this study under accession codes GSE75748, GSE203156 and HRA006197.
