# Supplementary material for: Large‐Scale Bioprinting of Human Epiblast‐Like Models Featuring Disc‐Shaped Morphogenesis and Gastrulation Events
Source: Adv Sci (Weinh). 2025 Jun 5;12(33):e05340. doi: 10.1002/advs.202505340 (PMC12412627; doi:10.1002/advs.202505340)
Supplement: Supplementary file 1 — Supporting Information [file ADVS-12-e05340-s003.docx]

Supporting Information

Large-Scale Bioprinting of Human Epiblast-Like Models Featuring Disc-Shaped Morphogenesis and Gastrulation Events

Yixue Luo, Liheng Luo, Ling Wang, Shanshan Yang, Hongan Ren, Shaojun Liang, Xiaoyu Wang, Yijun Su, Leqian Yu, Xiaoyue Wang*, Mingen Xu*, Rui Yao*


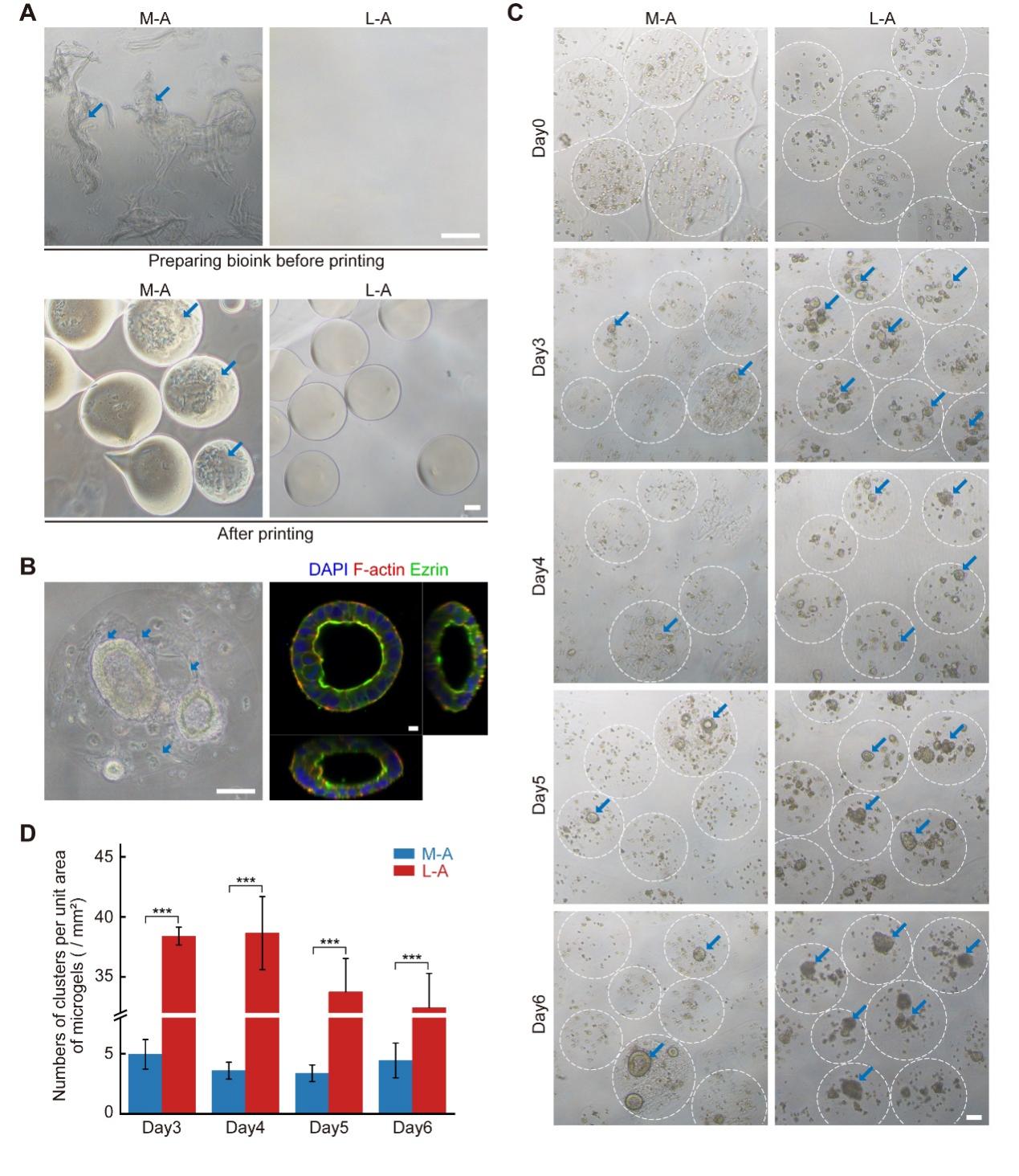


**Figure S1.** Optimization of the bioink’s bioactive component. A) Mixed bioink before and after printing. Blue arrows indicate floc-containing regions. Scale bar, 100 μm. B) Morphogenesis of cellular structures on day6 within M-A microgels. The left is optical microscope images for cavity-like structures with blue arrows denoting floc-like components, scale bar, 100 μm; the right is the immunofluorescence co-staining images of F-actin and Ezrin, scale bar, 25 μm. C) Optical microscope images showing the growth of human induced pluripotent stem cells (hiPSCs) within microgels containing different bioactive components. Blue arrows denote cell clusters. Scale bar, 100 μm. D) Statistic analysis of clustering efficiency. Data are presented as mean ± s.d. (n=3 independent fields of view). Data are statistically analyzed using independent-samples T test. *** means P < 0.001; no marking means no significant difference.
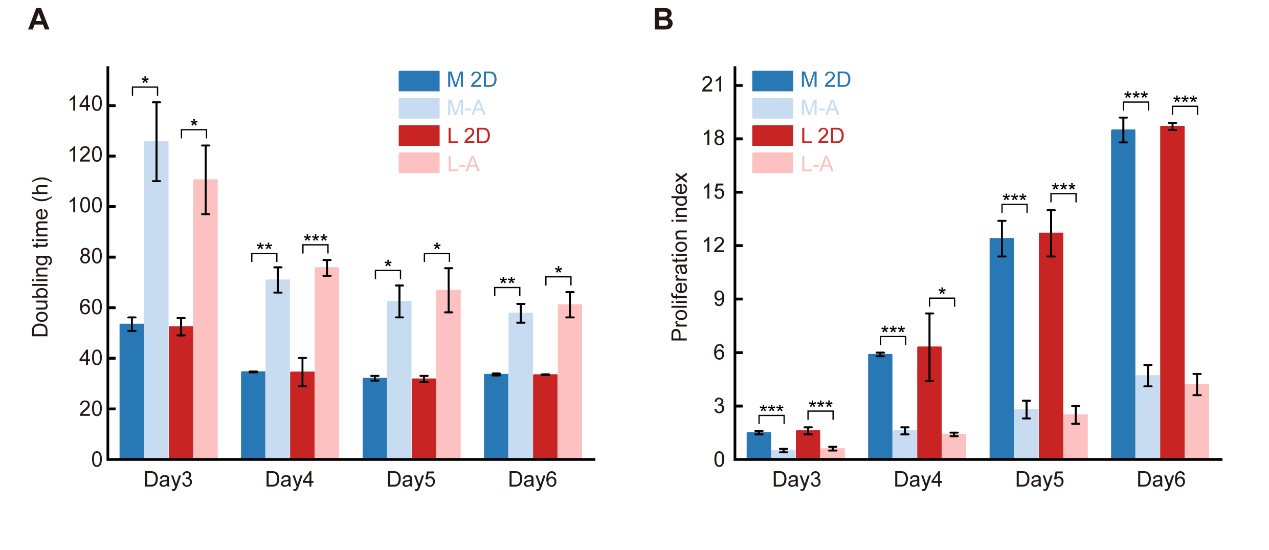


**Figure S2.** Proliferation rate of hiPSCs under different conditions. A, B) Comparison of cell doubling time A) and proliferation index B). M 2D and L 2D denote 2D plates coated with Matrigel and laminin-511E8F, respectively. Data are presented as mean ± s.d. (n=3 independent samples). Data are statistically analyzed using independent-samples T test. * means P < 0.05; **means P < 0.01; ***means P < 0.001; no marking means no significant difference.


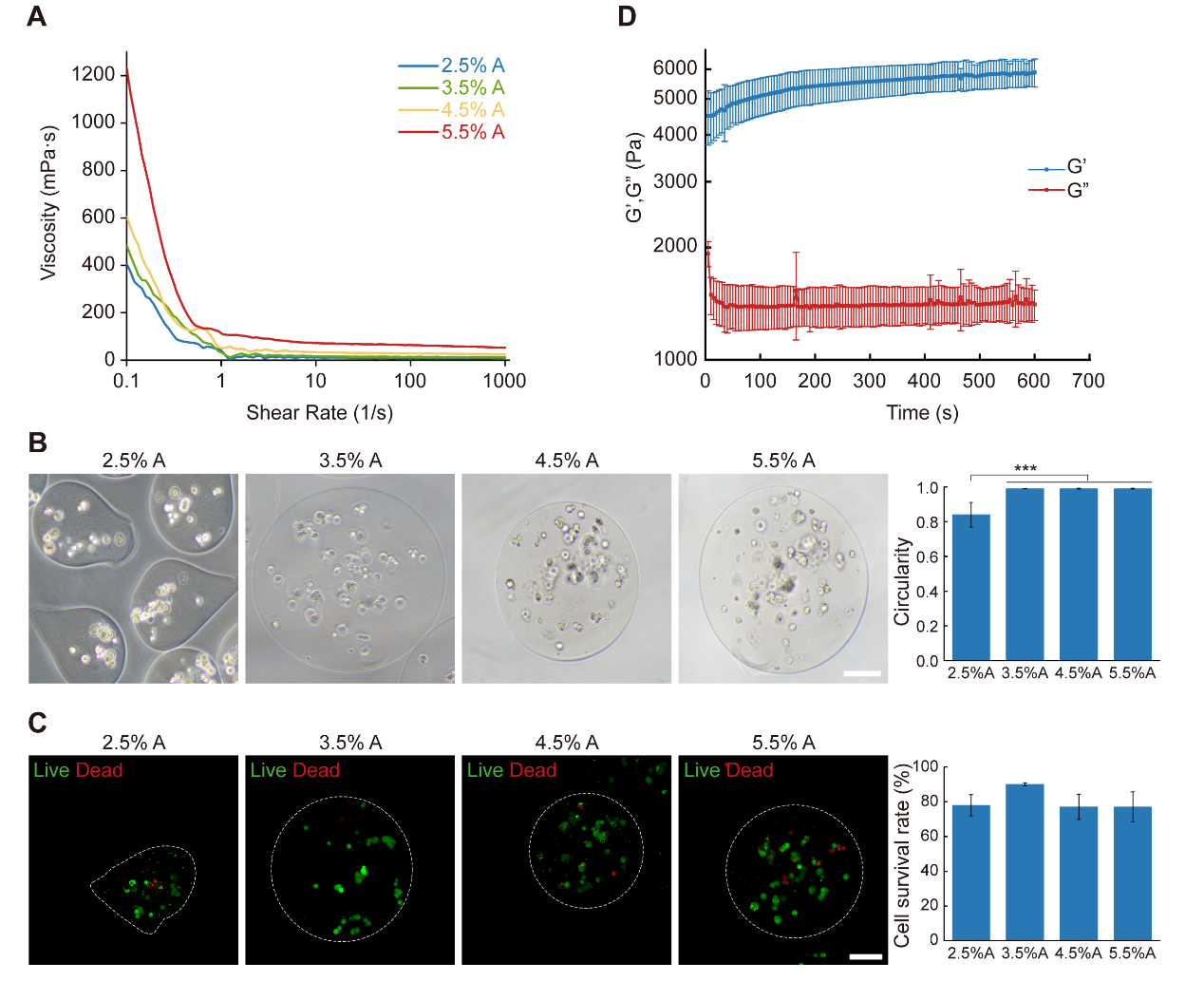


**Figure S3.** Optimization of the bioink’s backbone component. A) Shear-thinning properties of bioink at different concentrations of sodium alginate. B) Microgel circularity at different concentrations of sodium alginate. Scale bar, 100 μm. Data are presented as mean ± s.d. (n=20 microgels for circularity analysis). Data are statistically analyzed using one-way ANOVA; *** means P < 0.001; no marking means no significant difference. C) Cell survival rate at different concentrations of sodium alginate. Scale bar, 100 μm. Data are presented as mean ± s.d. (n=3 independent fields of view; at least 6 microgels per field of view). Data are statistically analyzed using one-way ANOVA; no marking means no significant difference. D) Viscoelastic characteristic of crosslinked L-A. G’ is the storage modulus, presenting the elasticity; G” is the loss modulus, presenting the viscosity. Data are presented as mean ± s.d. (n=3 independent samples).


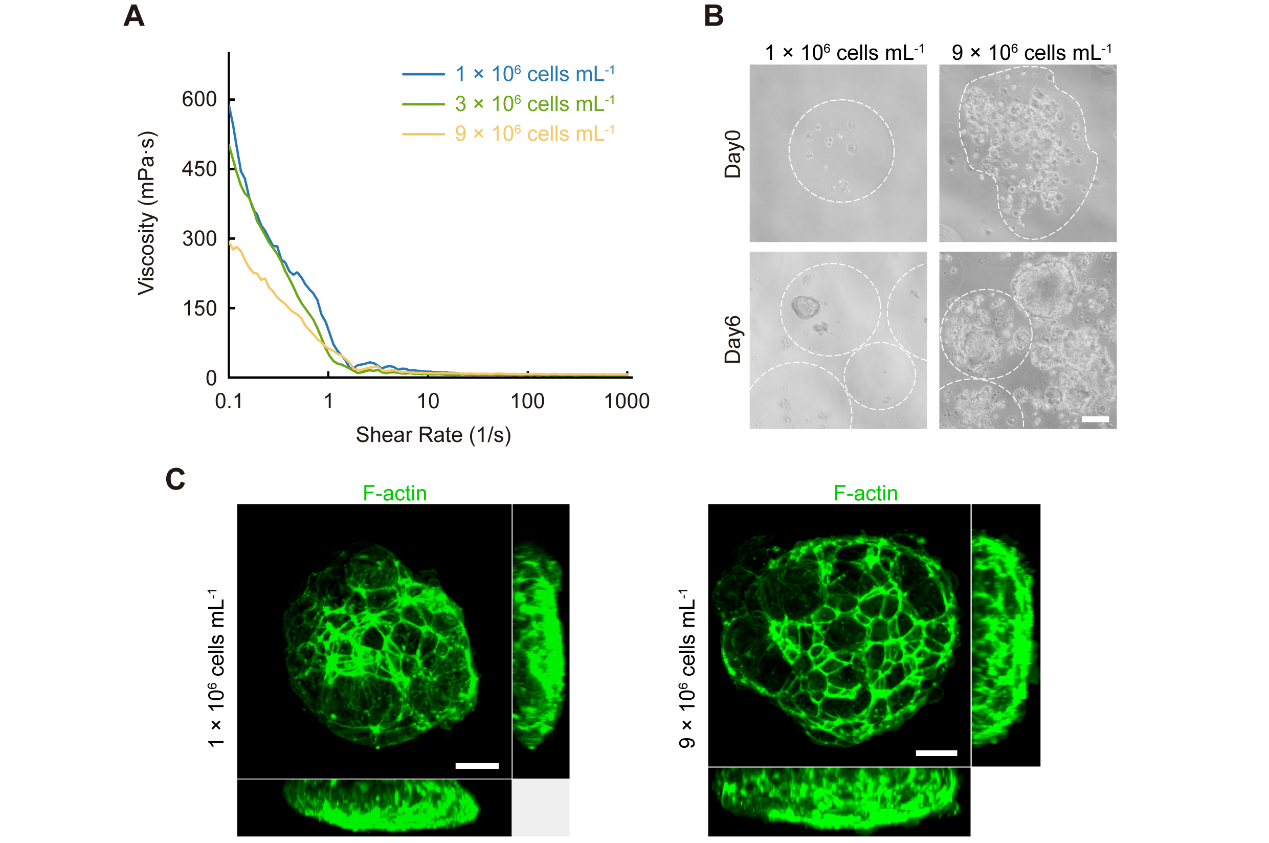


**Figure S4.** hiPSCs form disc-like structures at different initial cell densities. A) Shear-thinning properties of bioink at different initial cell densities. B) Optical microscope images of clusters within L-A microgels at different initial cell densities. White dashed lines denote the outlines of the microgels. Scale bar, 100 μm. C) Immunofluorescence images of F-actin in clusters generated from different initial cell densities on day6. Scale bar, 25 μm.


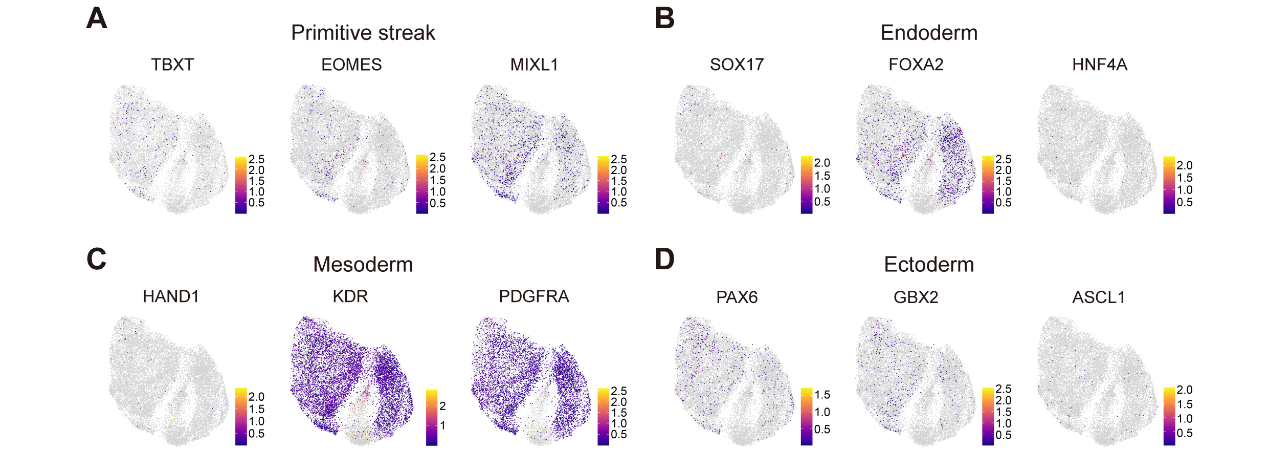


**Figure S5.** Gene expression of specific lineage markers. A-D) Specific marker expression of primitive streak A), endoderm B), mesoderm C) and ectoderm D).


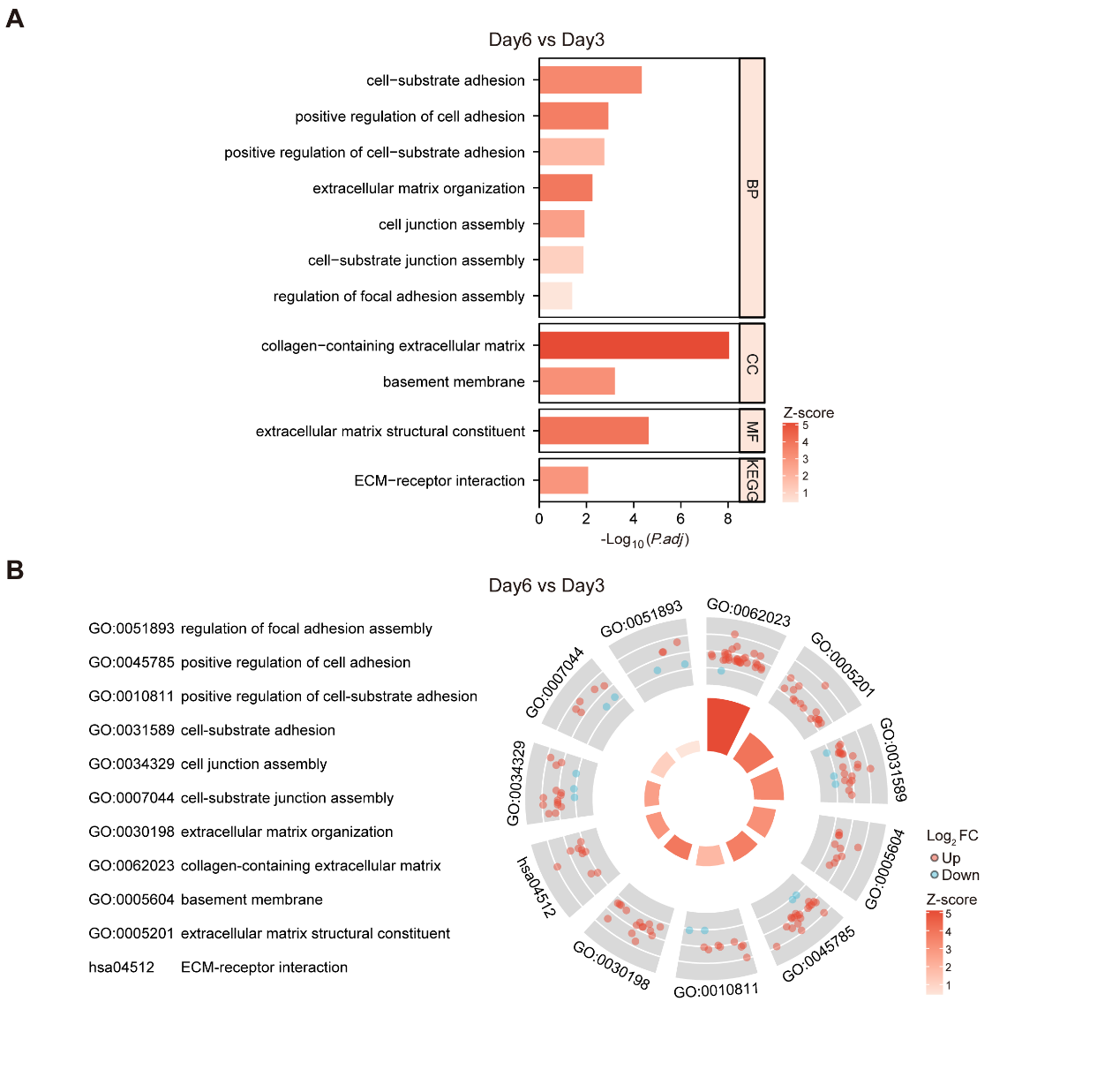


**Figure S6.** Alterations in cellular interaction with surroundings within L-A microgels. A, B) Chordal graph A) and loop graph B) depicting alterations in cellular interaction with surroundings for comparing samples on day6 versus day3.


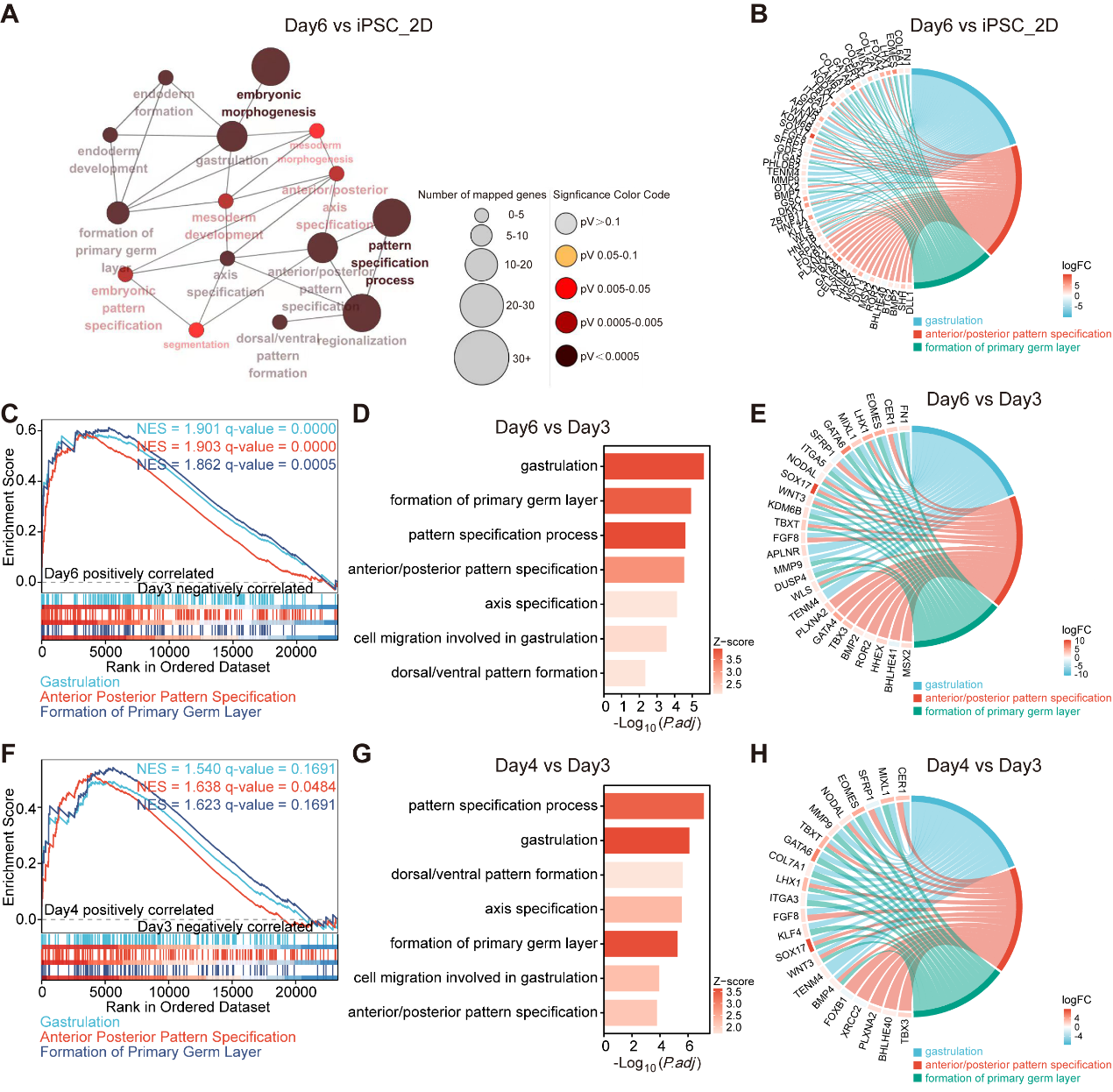


**Figure S7.** Epiblast-like structures tend to gastrulate from day3. A, B) GO enrichment of biological process A) and chordal graph depicting gastrulation-related biological processes and DEGs included in these terms B) for the comparison of samples on day6 versus iPSC_2D, Cytoscape 3.8.0 is used and Kappa Score is set to 0.4 in A). C-E) Representative plots of GSEA C), column chart depicting GO enrichment D) and chordal graph depicting gastrulation-related biological processes and DEGs included in these terms E) for the comparison of samples on day6 versus day3. F-H) Representative plots of GSEA F), column chart depicting GO enrichment G) and chordal graph depicting gastrulation-related biological processes and DEGs included in these terms H) for the comparison of samples on day4 versus day3.


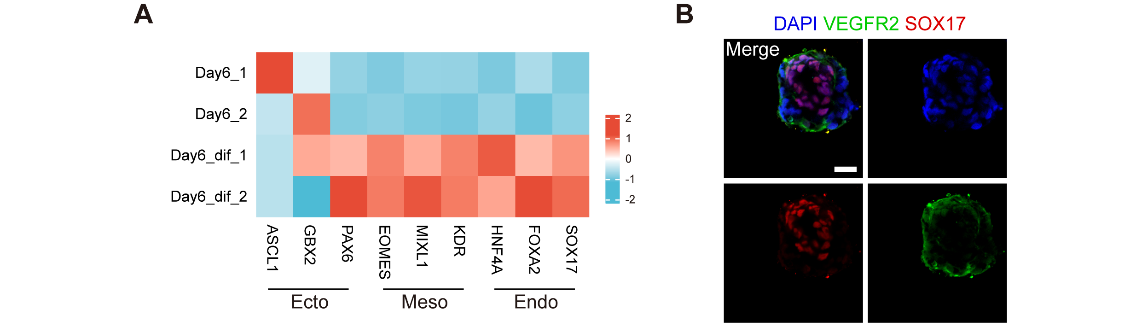


**Figure S8.** Differentiation potential of epiblast-like structures. A) A heatmap of representative gene expression for endoderm (Endo), mesoderm (Meso) and ectoderm (Ecto). B) Immunofluorescence co-staining images of VEGFR2 and SOX17. Scale bar, 25 μm.


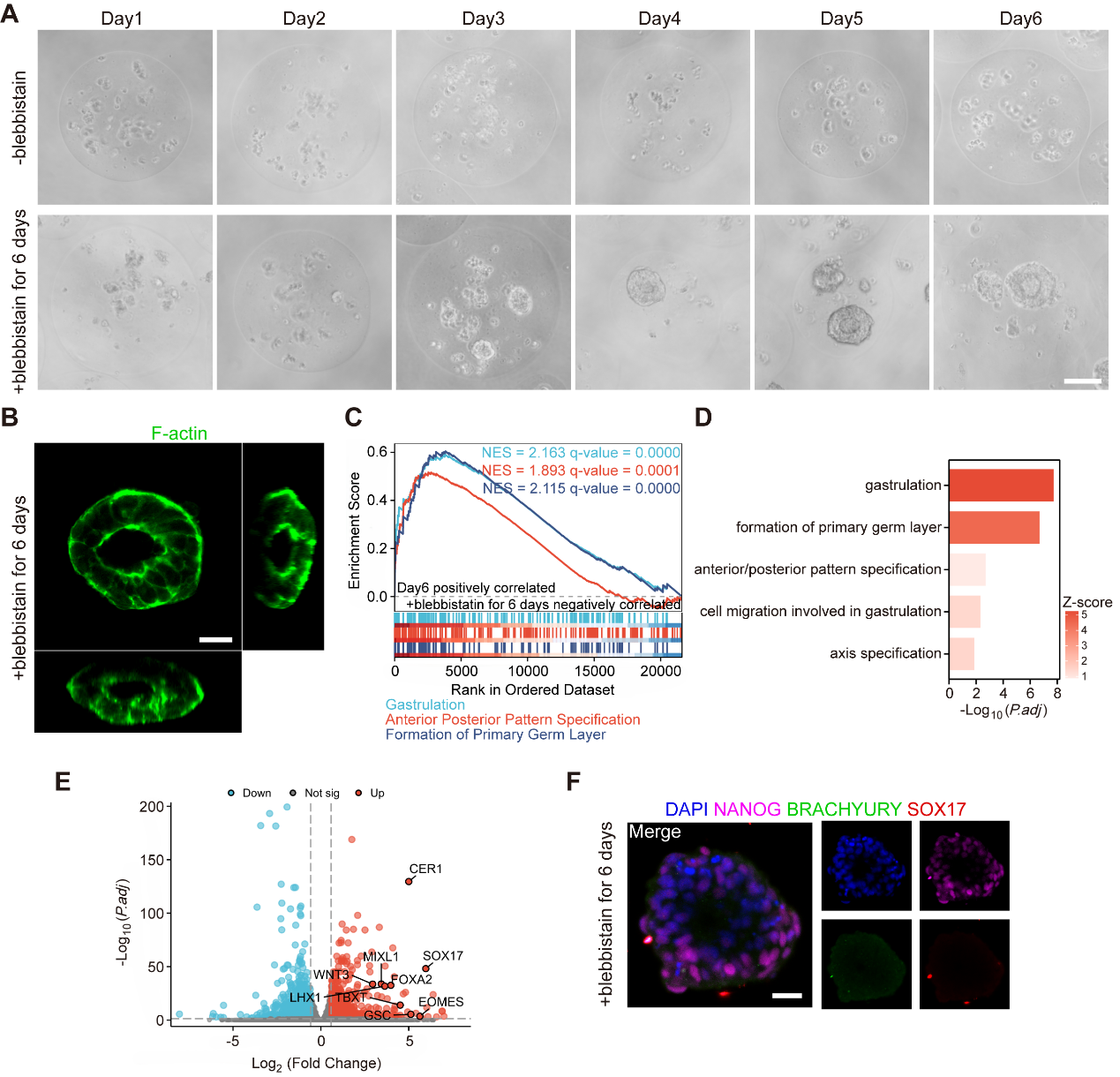


**Figure S9.** The effect of blebbistatin on hiPSCs within microgels. A) Optical microscope images of hiPSC clustering in the absence or persistent presence of blebbistatin within L-A microgels. Scale bar, 100 μm. B) Immunofluorescence images of F-actin in clusters on day6 in the persistent presence of blebbistatin. Scale bar, 25 μm. C-E) Representative plots of GSEA C), column chart depicting GO enrichment D) and volcano map depicting DEGs E) for the comparison of samples on day6 versus those in the persistent presence of blebbistatin on day6. F) Immunofluorescence staining images of NANOG, BRACHYURY and SOX17 for disc-like structures in the persistent presence of blebbistatin on day6. Scale bar, 25 μm.


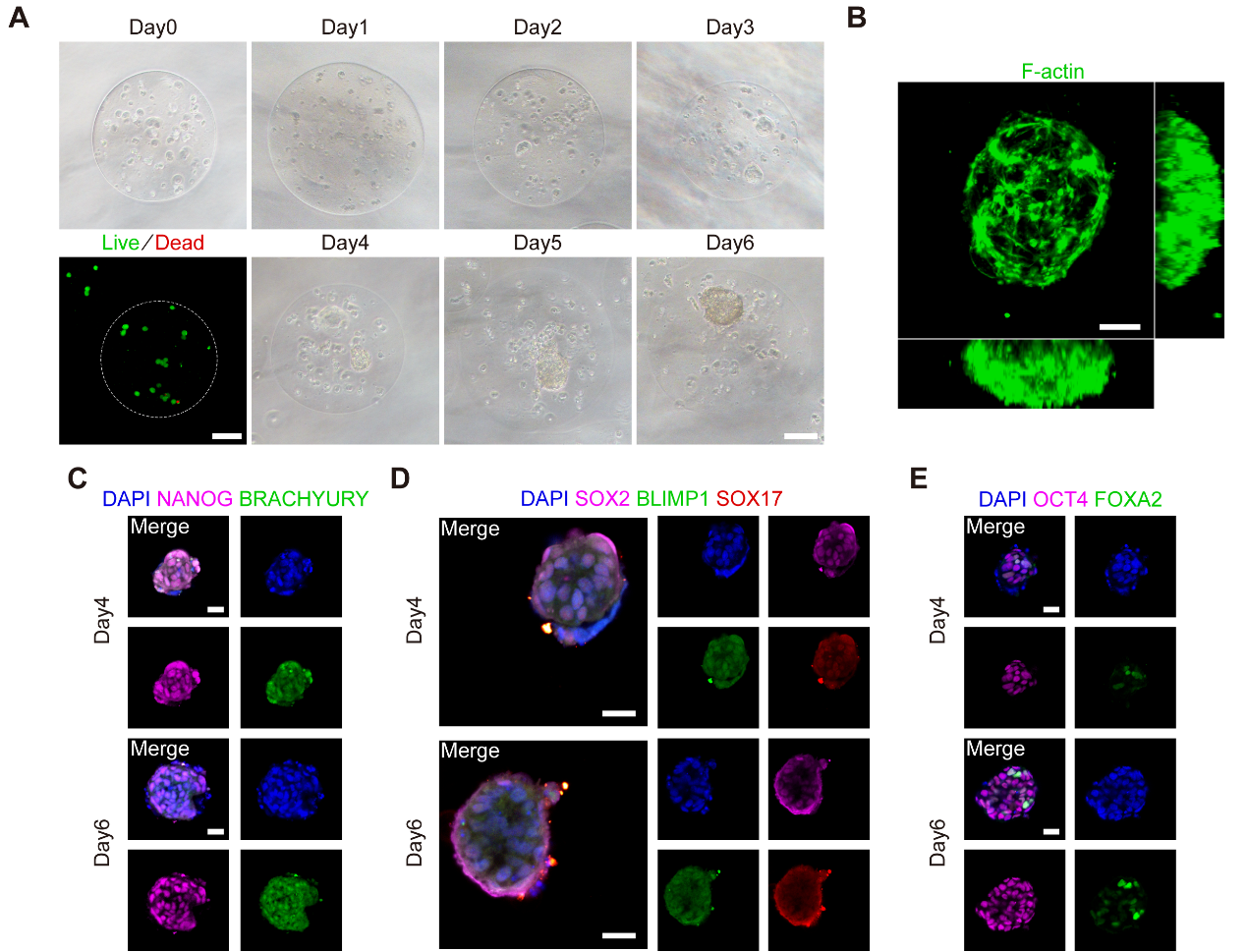


**Figure S10.** Human embryonic stem cells (hESCs) form epiblast-like tissues in A-L microgels. A) Optical microscope images of hESC clusters showing temporal morphology. Cell viability of hiPSCs on day0 is displayed in the lower left corner of A). Scale bar, 100 μm. B) Immunofluorescence images of F-actin for hESC clusters on day6. Scale bar, 25 μm. C-E) Immunofluorescence co-staining images of NANOG, BRACHYURY C), SOX2, BLIMP1, SOX17 D) and OCT4, FOXA2 E) for disc-like tissues. Scale bar, 25 μm.


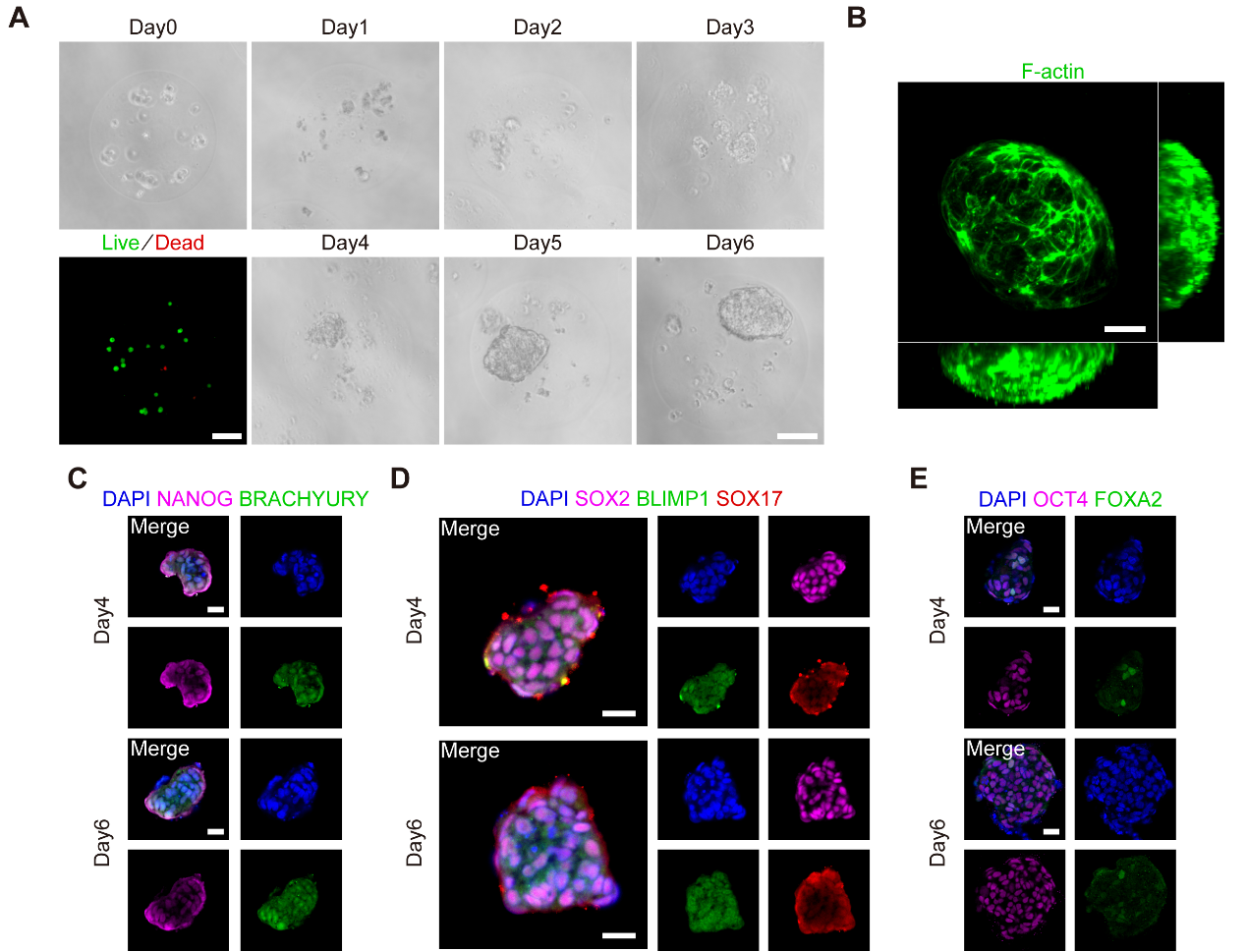


**Figure S11.** hiPSCs form epiblast-like tissues in A-L microgels using an alternative self-renewing medium. A) Optical microscope images of hiPSC clusters cultured in an alternative self-renewing medium showing temporal morphology. Cell viability of hiPSCs on day0 is displayed in the lower left corner of A). Scale bar, 100 μm. B) Immunofluorescence images of F-actin for clusters cultured in an alternative self-renewing medium on day6. Scale bar, 25 μm. C-E) Immunofluorescence co-staining images of NANOG, BRACHYURY C), SOX2, BLIMP1, SOX17 D) and OCT4, FOXA2 E) for disc-like tissues cultured in an alternative self-renewing medium. Scale bar, 25 μm.

**
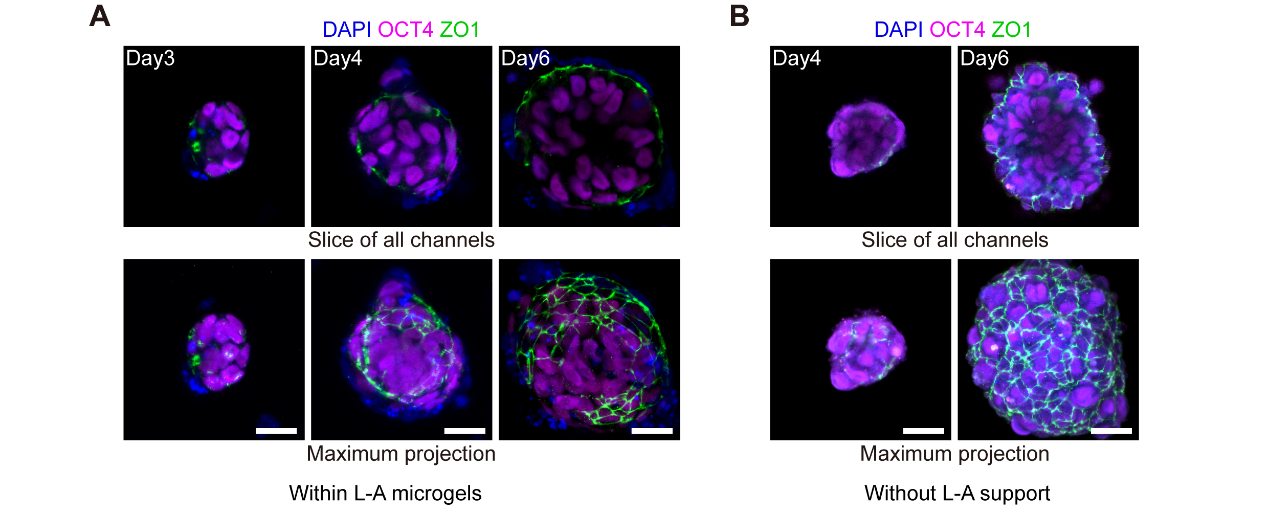
**

**Figure S12.** Cell junction in clusters within L-A microgels and without A-L support. A, B) Immunofluorescence co-staining images of OCT4 and ZO1 in clusters within L-A microgels A) or without A-L support B). Scale bar, 25 μm.


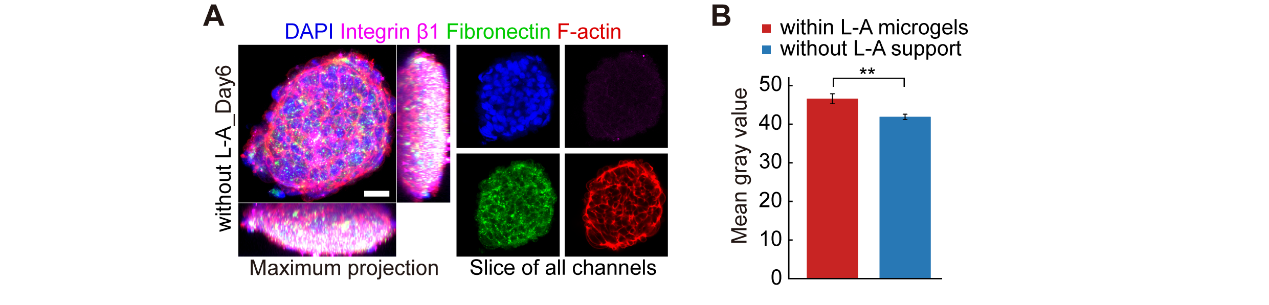


**Figure S13.** Extracellular matrix changes between clusters within L-A microgels and without L-A support. A) Immunofluorescence co-staining images of Fibronectin, Integrin β1 and F-actin in clusters without L-A support. B) Comparison of mean gray value for Integrin β1 within L-A microgels and without A-L support. Data are presented as mean ± s.d. (n=3 independent fields of view). Data are statistically analyzed using independent-samples T test. ** means P < 0.01.
